# The complement C3-microglial axis in depression of Parkinson's disease: from mechanism to therapeutic intervention

**DOI:** 10.1016/j.ebiom.2026.106325

**Published:** 2026-06-09

**Authors:** Qiao Yin, Mengyang Ding, Yurui Tang, Yuwan Qi, Yuan Qin, Hong Jin, Yang Li, Jili Bao, Shuyang Ma, Ying Li, Haozhe Ding, Xinyu An, Enyou Qiao, Yan Tang, Qilin Zhang, Linna Wang, Jianfeng Shao, Jianfeng Feng, Li-Fang Hu, Jing Wang, Pan Fang, Weifeng Luo, Qifei Cong

**Affiliations:** a Department of Neurology and Clinical Research Center of Neurological Disease, The Second Affiliated Hospital of Soochow University, Suzhou, 215004, China; b Institute of Molecular Enzymology, School of Life Sciences, Suzhou Medical College, Soochow University, Suzhou, 215123, China; c Institute of Science and Technology for Brain-Inspired Intelligence, Fudan University, Shanghai, 201203, China; d Jiangsu Key Laboratory of Drug Discovery and Translational Research for Brain Diseases, Institute of Neuroscience, Soochow University, Suzhou, 215123, China; e Department of Neurology, Huzhou Central Hospital, The Fifth School of Clinical Medicine of Zhejiang Chinese Medical University, Huzhou, 313000, China; f Lanzhou Biotechnique Development Co., Ltd., Lanzhou, Gansu, 730000, China; g Department of Neurology, The Third People's Hospital of Zhangjiagang City, Suzhou, 215600, Jiangsu, China; h State Key Laboratory of Chemical Biology, Shanghai Institute of Organic Chemistry, Chinese Academy of Sciences, Shanghai, 200032, China; i Biomedical Basic Research Center (BBRC) of Jiangsu, Soochow University, Suzhou, 215123, China; j Department of Nephrology, The Second Affiliated Hospital of Soochow University, Suzhou, 215004, China; k Institute of Neurological Diseases, Soochow University-Suzhou Blue Cross Brain Hospital, Soochow University, Suzhou, 215123, China; l School of Life Sciences, MOE Key Laboratory of Geriatric Diseases and Immunology, Suzhou Medical College of Soochow University, Soochow University, Suzhou, 215123, China

**Keywords:** Depression in Parkinson's disease, Plasma proteomics, C3–C3aR signalling, Microglia, Synaptic pruning, Botulinum neurotoxin A

## Abstract

**Background:**

Depression is a common and early non-motor symptom of Parkinson's disease (PD) with significant sexual dimorphism, yet its underlying molecular mechanisms remain poorly understood. This study aimed to elucidate the sex-specific plasma proteomic profiles of depression in patients with PD (DPD) and to investigate the role of complement-mediated synaptic pruning in its pathophysiology.

**Methods:**

Plasma proteomic analysis was performed on data from the Parkinson's Progression Markers Initiative (PPMI) and an independent validation cohort, stratified by sex. Functional enrichment analyses identified dysregulated pathways. A chronic MPTP/probenecid-induced mouse model of PD was used to validate findings. Behavioural tests assessed motor and depressive-like phenotypes. Proteomic, biochemical, and imaging techniques were used to evaluate protein expression, synapse density, and microglial phagocytosis. The therapeutic mechanism of Botulinum Neurotoxin A (BoNT/A) on DPD was investigated in wild-type, *C3*^*−/−*^ and *C3aR*^*−/−*^ mice and in microglial cultures.

**Findings:**

Proteomic profiling revealed both conserved complement-driven immune dysfunction and profound sex-divergent molecular perturbations underlying PD and DPD. Complement and coagulation cascades were consistently upregulated in both sexes. In MPTP-treated male and female mice, hippocampal complement components (C1Q, C3, C3aR) and downstream signalling (p-STAT3, p-P65) were elevated, accompanied by microglial synapse phagocytosis and depressive-like behaviours. Genetic deletion of *C3* rescued both MPTP-induced motor and depressive-like behavioural deficits and prevented hippocampal synaptic loss associated with microglial synaptic engulfment. BoNT/A treatment alleviated depressive-like behaviours and reduced microglial synaptic engulfment in an MPTP model; these therapeutic effects were abolished in *C3*^*−/−*^ and *C3aR*^*−/−*^ mice. Single-cell RNA sequencing and in vitro phagocytosis assay confirmed that BoNT/A modulated phagocytosis-related microglial subclusters.

**Interpretation:**

DPD exhibits distinct sex-specific immune signatures, with convergent complement pathway activation driving microglial synaptic pruning and depressive symptoms. The antidepressant effect of BoNT/A is mediated through inhibition of the C3–C3aR signalling axis. These findings highlight the potential for sex-stratified diagnostics and complement-targeted therapies for depression in patients with PD. A key limitation is that our clinical analyses were constrained by limited validation cohort sizes, and mechanistic studies were limited to male mice, which may restrict the generalisability of our findings to female populations.

**Funding:**

National Natural Science Foundation of China, Key Project of the Natural Science Foundation of Jiangsu Provincial Higher Education Institutions, Project of Biomedical Basic Research Center (BBRC) of Jiangsu, Clinical Research Center of Neurological Disease in The Second Affiliated Hospital of Soochow University, Project of MOE Key Laboratory of Geriatric Diseases and Immunology, Jiangsu Key Laboratory of Drug Discovery and Translational Research for Brain Diseases; The Lingang Laboratory fund; Shanghai Science and Technology Innovation Sailing Special Project, and Shanghai Municipal Science and Technology Major Project; Zhejiang Provincial Natural Science Foundation of China.

**Research in context**

**Evidence before this study**

Epidemiological studies have established significant sexual dimorphism in Parkinson's disease (PD). Notably, while men have a higher incidence of PD, women with PD are disproportionately affected by neuropsychiatric symptoms such as depression and anxiety. Preclinical and clinical research have separately implicated immune dysregulation, including complement system activation, in PD pathophysiology and suggested that Botulinum Neurotoxin A (BoNT/A) may have antidepressant properties. However, a major gap existed in understanding the sex-specific molecular mechanisms linking PD pathophysiology to comorbid depression. Furthermore, it remained unclear whether the emerging pathway of complement-mediated microglial synaptic pruning was a key driver of depressive symptoms in patients with PD, and if so, whether it represented a mechanistic target for interventions like BoNT/A.

**Added value of this study**

This study provides multi-level evidence that significantly advances the field. First, through proteomic profiling of clinical cohorts (PPMI and an independent validation cohort), we systematically define distinct, sex-specific plasma protein signatures associated with depression in patients with PD (DPD). Compared with HC, upregulated differentially expressed proteins (DEPs) in both PD and DPD showed shared enrichment of complement activation and immune response activation in both males and females. In contrast, downregulated DEPs exhibited significant sexual dimorphism: female downregulated DEPs were associated with metabolic and neurodegenerative pathways, while male downregulated DEPs were linked to lipoprotein and cholesterol remodelling. Furthermore, in the DPD versus PD comparison, DPD-specific dysregulated molecular changes were enriched in gut immunity and ER stress in males but were minimal in females. Second, we identify the complement C3–C3aR pathway as a convergent, functionally critical mechanism. Using a chronic MPTP/probenecid mouse model of PD with depression, we demonstrate that C3–C3aR signalling is upregulated in the hippocampus, leading to excessive microglial phagocytosis of synapses and the manifestation of depressive-like behaviours. Genetic deletion of *C3* rescued these effects. Third, we elucidate a specific mechanism of action for BoNT/A. We demonstrate that BoNT/A alleviates depressive-like behaviours in the PD model by inhibiting C3–C3aR-dependent microglial synaptic engulfment. This therapeutic effect was abolished in *C3*- and *C3aR*-deficient mice, establishing a direct causal link.

**Implications of all the available evidence**

The evidence advocates for sex-stratified diagnostic and therapeutic strategies for depression in patients with PD. The C3–C3aR pathway is a promising mechanistic target for treatment. Our findings provide a clear biological rationale for BoNT/A's antidepressant effect, informing its potential application. Future research should validate these sex-specific biomarkers in larger cohorts and explore complement modulation as a basis for personalised therapy in PD psychiatry.

## Introduction

Parkinson's disease (PD), the second most prevalent neurodegenerative disorder, displays significant sexual dimorphism in its epidemiology.^1^ Males exhibit a higher incidence and prevalence of PD^2^; however, female patients with PD experience a disproportionately increased risk of developing depression, a common and consequential comorbidity that can often serve as an early prodromal indicator.^3^, ^4^, ^5^, ^6^ This sex-specific susceptibility in PD, along with its psychiatric manifestations,^7^^,^^8^ highlights the limitations of the current uniform clinical approach that predominantly relies on subjective symptom evaluations and neglects the distinct underlying pathophysiological differences.^9^, ^10^, ^11^

While extensive proteomic investigations have catalogued protein alterations in various PD biospecimens,^12^, ^13^, ^14^ such as brain tissue,^15^ blood,^16^, ^17^, ^18^, ^19^ and cerebrospinal fluid (CSF),^20^^,^^21^ there has been insufficient focus on two critical dimensions: the molecular basis of depressive symptoms in patients with PD and the influence of sex on this profile. Therefore, it is imperative to advance from purely epidemiological observations to elucidate the sex-specific proteomic mechanisms underpinning depression in PD. Such insights are crucial for developing sex-stratified diagnostic biomarkers and personalised therapeutic strategies for this patient population.

Given these pronounced clinical differences, elucidating the underlying mechanisms of depression in PD—particularly how they may be influenced by sex—becomes essential. Currently, the mechanisms underlying depression in patients with PD are not yet fully understood. While psychological factors play a role, it appears that psychosocial factors and disability are not the predominant determinants of depressive disorders in individuals with PD.^22^, ^23^, ^24^ Accumulating evidence has implicated a spectrum of pathological changes, including disruptions in brain structure and functional connectivity,^25^, ^26^, ^27^, ^28^ neuronal circuitry,^29^^,^^30^ neurotransmitter signalling,^31^, ^32^, ^33^, ^34^, ^35^, ^36^ synaptic plasticity,^37^^,^^38^ gut microbiota,^39^ autophagy,^40^^,^^41^ and neuroinflammation.^42^, ^43^, ^44^, ^45^, ^46^ Among these, dysregulated neuroinflammation has emerged as a particularly critical hub linking neurodegeneration and affective symptoms, with the complement system increasingly recognised as a key mediator in this process.^5^^,^^47^ The complement cascade, classically part of the innate immune response, is now recognised for its significant functions in neurodevelopment, and the pathophysiology of neuropsychiatric and neurodegenerative disorders.^48^, ^49^, ^50^, ^51^ Research indicates that murine models of PD, whether induced by α-synuclein preformed fibrils (PFF) or overexpression of the A53T α-synuclein variant, demonstrate pronounced activation of both complement and coagulation cascades.^52^ However, the roles of complement proteins and microglia in PD have not been extensively studied. Furthermore, it remains unclear whether these factors contribute to synapse loss or the onset of depressive symptoms associated with PD.

Currently, a range of therapeutic strategies, including pharmacological, psychotherapeutic, and lifestyle interventions, has been proposed to target depression in patients with PD. Botulinum neurotoxin A (BoNT/A), produced by gram-positive bacteria *Clostridium botulinum*, binds to synaptic vesicle protein 2 (SV2) receptors, enters the cytosol via endocytosis, and cleaves SNAP-25 to inhibit acetylcholine release at synaptic junctions.^53^^,^^54^ It is well-established for the treatment of various neurological disorders, including dystonia, spasticity, headaches, hemifacial spasm, essential tremor, motor tics, hyperhidrosis, and sialorrhoea.^55^, ^56^, ^57^ Recent clinical trials have also demonstrated its benefits for major depression,^58^^,^^59^ and depression in patients with PD.^60^, ^61^, ^62^ Emerging evidence suggests that BoNT/A may exert its effects by modulating microglial activation and neuroinflammatory pathways.^63^, ^64^, ^65^, ^66^ However, the specific mechanisms by which BoNT/A alleviates Parkinsonism-related depression remain incompletely understood. A key question is whether its antidepressant effects are mediated by the suppression of complement-driven microglial phagocytosis of synapses.

In this study, we aimed to systematically elucidate the sex-specific plasma proteomic profiles and the role of complement-mediated immune mechanisms in patients with PD with depressive symptoms (DPD). By analysing data from the Parkinson's Progression Markers Initiative (PPMI) dataset alongside an independent validation cohort, we identified significant sexual dimorphisms in proteomic signatures and pathway enrichment, particularly involving immune activation, coagulation cascades, and complement pathways. Based on these clinical findings, we further investigated the role of complement component C3 and its receptor C3aR in microglia-mediated synaptic pruning and depressive-like behaviours in a chronic MPTP/probenecid-induced mouse model of PD. Additionally, we evaluated the therapeutic potential of BoNT/A for ameliorating these depressive phenotypes by modulating complement-dependent microglial phagocytosis.

## Methods

### Sex as a biological variable

Our human studies are reported for both sexes. Considering the role of complement-mediated inflammation in both female and male patients with DPD, we utilised both male and female mice to create a chronic MPTP-induced Parkinsonian model to explore the complement cascade's involvement in PD with comorbid depression.

### Proteomic data analysis from PPMI for Parkinson's disease

The proteomic data used in this study were obtained from the Parkinson's Progression Markers Initiative (PPMI, Project ID: 196), generated by AbbVie in collaboration with the AMP PD Proteomics Working Group using the Olink® Explore 1536 platform. The dataset included 920 plasma samples analysed via Proximity Extension Assay (PEA) technology, which quantifies relative protein abundance through dual antibodies tagged with oligonucleotides. Raw sequencing counts were internally normalised and converted to Normalised Protein Expression (NPX) values on a log_2_ scale. Each sample included three internal quality controls—incubation, extension, and amplification controls—and three external controls (Plate Control, Sample Control, and Negative Control) to ensure technical consistency. Intensity normalisation was applied across plates to achieve inter-plate calibration.

According to the Olink Certificate of Analysis (November 2022), >90% of samples across all panels passed QC, with >94% of proteins detected, an average intra-assay coefficient of variation (CV) below 10%, and an inter-assay CV below 22%.

Data preprocessing followed Olink's recommended standards. Samples flagged with “WARN” in the QC_WARNING column were excluded, as were proteins with ≥50% missing values (MISSINGFREQ ≥0.5). Remaining missing NPX values were imputed by protein-wise mean substitution. Data from the four Olink panels were merged into a long-format table, and only baseline samples were retained. Depression in PD (DPD) was defined as GDS ≥9, while non-depressed PD was defined as GDS ≤4; samples with intermediate scores^5^, ^6^, ^7^, ^8^ were excluded from the analysis to enhance the contrast between groups and ensure the clarity of the molecular signatures. Differential expression analysis was performed using a two-sample t-test. A threshold of |log_2_FC| > 0.3785 (≈1.3-fold) and P < 0.05 was pre-specified according to widely used standards in Olink proteomic studies. Candidate proteins identified under these criteria were subsequently validated experimentally and characterised mechanistically.

### Functional enrichment analysis

Significantly differentially expressed proteins were subjected to Gene Ontology (GO) Biological Process enrichment analysis using the clusterProfiler package in R. Protein symbols were converted to Entrez Gene IDs using the org.Hs.eg.db database. The significance thresholds were set at p < 0.05. Enrichment results were visualised using −log_10_(p) values, and pathway interpretation was restricted to biologically coherent terms to avoid overgeneralization.

### Animals and treatments

Wild-type C57BL/6J mice, *C3* knockout (*C3*^*−/−*^) mice and *C3aR* knockout (*C3aR*^*−/−*^) mice were used in this study. Male and Female C57BL/6J mice (RRID: IMSR_GPT: N000013), aged 12 weeks, were obtained from GemPharmatech Biotechnology (Nanjing, China). The *C3*^*−/−*^ mice (The Jackson Laboratory, RRID: IMSR_JAX: 029661) and *C3aR*^*−/−*^ mice (Gempharmatech Biotechnology, Nanjing, RRID: IMSR_GPT: T006106) were generously provided by Professors Weiguo Hu from Fudan University and Chao Yan from Nanjing University, respectively. All mice were maintained in a specific pathogen-free (SPF) environment, under a controlled 12-h light/dark cycle, with access to adequate food and water. All animal experiments in this study involving mice complied with animal welfare regulations and were approved by Soochow University's Animal Ethics Committee (Approval No. SUDA20240911A10).

We employed the MPTP-induced mouse model to induce Parkinson's disease, adapting the protocol from previous studies.^67^ Mice received intraperitoneal injections of 1-methyl-4-phenyl-1, 2, 3, 6-tetrahydropyridine (MPTP) at a dosage of 20 mg/kg (Sigma–Aldrich, Cat# M0896). This was followed by an intraperitoneal administration of probenecid (250 mg/kg, Aladdin, Cat# P129440) 1 h later.^67^ In this study, all MPTP-treated mice refer to MPTP/probenecid-treated mice, and the group abbreviation is uniformly simplified as MPTP throughout the manuscript. Post-modelling, a series of behavioural assays were conducted to evaluate motor capabilities and depressive behaviours in the subjects. For BoNT/A intervention, the mice were subjected to bilateral injections of BoNT/A (10 U/kg, Lanzhou Institute of Biological Products Co., Ltd, Cat. No. S10970037) targeting the facial musculature.

### Behavioural tests

#### Open field test

Spontaneous locomotor activity was evaluated in an opaque acrylic open field apparatus (40 × 40 × 40 cm). Each mouse was positioned at the centre of the arena, and its movements were monitored for 10 min using an automated video tracking system (XR-XZ301, Shanghai XinRuan Information Technology Co., Ltd., China). The total distance travelled during this session was quantified as a metric of general locomotor activity.

#### Pole test

A pole test was performed to assess motor coordination and bradykinesia in mice, following established methodologies.^68^ The apparatus consisted of a 52 cm vertical wooden rod (1 cm in diameter), with a 2.5 cm diameter wooden sphere affixed to the apex. To enhance grip and minimise slippage, the rod was wrapped in gauze. During the assessment, each mouse was carefully positioned head-down at the top of the pole, with its hind limbs resting on the sphere. The time taken for each mouse to descend to the base was recorded. Each subject underwent three trials, with a minimum intertrial interval of 30 min. The mean descent time from these trials was subsequently analysed for statistical significance.

#### Rotarod test

Motor coordination and balance were assessed using a rotarod apparatus (XR-6C, Shanghai XinRuan Information Technology Co., Ltd., China), following a modified protocol based on previously established methodologies.^69^ Mice underwent training over three consecutive days. On Day 1, subjects were placed on a constant-speed rod set at 4 rpm for 5 min per trial, with each mouse undergoing three trials. For Days 2 and 3, an accelerating protocol was implemented, increasing speed from 4 to 40 rpm over a 5-min duration, with a minimum of three trials conducted each day. Testing occurred on Day 4, where mice participated in three trials under the same accelerating conditions, interspersed with at least 30-min intervals between trials. A trial concluded when a mouse fell from the rod, and the average latency to fall was recorded for each subject.

#### Forced swimming test

To assess behavioural despair, individual mice were submerged in a transparent cylindrical tank (20 × 30 cm) containing water to a depth of 22 cm, maintained at a temperature range of 22–24 °C. A front-facing camera captured their behaviour throughout a 6-min trial. Immobility was quantified as the time spent inactive, excluding movements necessary to elevate the head above the water's surface. This parameter was analysed during the final 4 min of the session using behavioural analysis software (XR-XQ202, Shanghai XinRuan Information Technology Co., Ltd., China).

#### Tail suspension test

Mice were suspended by the tail with medical adhesive tape applied about 1 cm from the tail tip, connected to a hook positioned 50 cm above the ground. Each experimental session lasted 6 min and was captured using a front-facing camera. Immobility time, operationally defined as the absence of movement apart from those necessary for postural maintenance, was analysed during the final 4 min of the recording using the software (XR-XQ202, Shanghai XinRuan Information Technology Co., Ltd., China).

#### Sucrose preference test

The sucrose preference test was executed according to a protocol modified from a prior study.^70^^,^^71^ Initially, from Day 1 (19:00) to Day 2 (19:00), mice had access to two bottles containing a 1% sucrose solution. On Day 2 to Day 3, one of the sucrose bottles was substituted with tap water, and at 07:00 on Day 3, the positions of the bottles were interchanged to eliminate potential side bias. From Day 3 (19:00) until Day 4 (19:00), the mice were subjected to a deprivation period of both food and water. During the testing phase, which spanned from Day 4 to Day 5, each cage was provided with one bottle of 1% sucrose solution and one bottle of water, with the bottles being repositioned again at 07:00 on Day 5. Liquid consumption was quantified before testing and 24 h after testing. Sucrose preference was computed using the formula: Sucrose preference (%) = [sucrose intake/(sucrose intake + water intake)] × 100.

### Western blotting

Proteins were extracted using RIPA lysis buffer (Beyotime Cat# P0013C, Shanghai) supplemented with protease and phosphatase inhibitors, separated by SDS-PAGE, and transferred to PVDF membranes. After blocking with 5% skim milk, membranes were incubated overnight at 4 °C with the following primary antibodies. All commercial antibodies used in this study were validated for specificity in the reported applications, and RRIDs were provided. TH (Cell Signalling Technology Cat# 58844, USA, 1:1000, RRID: AB_2744555), C1qa (ABclonal Cat# A24519, Wuhan, 1:1000, RRID: AB_3716887), C3 (MP Biomedical Cat# 855730, USA, 1:1000, RRID: AB_3716888), C3 (Proteintech Cat# 66157-1-Ig, Wuhan 1:2500, RRID: AB_2881553), C3aR (ABclonal Cat# A6361, Wuhan, 1:1000, RRID: AB_2766963), p-STAT3 (Abcam Cat# ab32143, UK, 1:5000, RRID: AB_2286742), STAT3 (Cell Signalling Technology Cat# 4904, USA, 1:2000, RRID: AB_331269), p-P65 (Affinity Biosciences Cat# AF3387, USA, 1:1000, RRID: AB_2834818), P65 (Affinity Biosciences Cat# AF1017, USA, 1:1000, RRID: AB_2835385), NF-κB-P65 (phospho S536) (ImmunoWay Cat# YP0191, USA, 1:2000, RRID: AB_2893502), and GAPDH (MesGen Biotechnology Cat# MAN1002, Shanghai, 1:2000, RRID: AB_3716889). After TBST washing, membranes were incubated with HRP-conjugated secondary antibodies—Goat anti-rabbit IgG (H+L)-HRP (MesGen Biotechnology Cat# MAN4001, Shanghai, 1:2500, RRID: AB_3716890), Goat anti-mouse IgG (H+L)-HRP (MesGen Biotechnology Cat# MAN4002, Shanghai, 1:2000, RRID: AB_3716891), or Donkey anti-goat IgG (H+L)-HRP (Jackson ImmunoResearch Labs Cat# 705-035-003, USA, 1:1000, RRID: AB_2340390)—and visualised using an ECL detection system (Tanon, China).

### Immunofluorescence staining

For immunofluorescence staining, mice were anaesthetised with Avertin (0.5 mg/g) and perfused transcardially with phosphate-buffered saline (PBS) followed by 4% paraformaldehyde (PFA) in PBS. Brains were then post-fixed in 4% PFA overnight and subsequently cryoprotected in 30% sucrose prepared in PBS. Coronal brain sections (30 μm thick) were obtained using a sliding freezing microtome (SM 2010R, Leica, Germany). After washing the sections three times each with PBS and PBST (PBS containing 0.25% Triton X-100), blocking was performed for 1 h using 5% normal goat serum in PBST. Sections were then incubated overnight at 4 °C with primary antibodies, including: chicken anti-Iba1 (Synaptic Systems Cat# 234 009, 1:1000, RRID: AB_2891282), rat anti-CD68 (Abcam Cat# ab53444, 1:1000, RRID: AB_869007), guinea pig anti-VGLUT1 (Synaptic Systems Cat# 135 318, 1:1000, RRID: AB_2924948), chicken anti-Homer1 (Synaptic Systems Cat# 160 019, 1:1000, RRID: AB_3662614), guinea pig anti-VGAT (Synaptic Systems Cat# 131 308, 1:1000, RRID: AB_2832243), and mouse anti-Gephyrin (Synaptic Systems Cat# 147 021, 1:1000, RRID: AB_2232546). All commercial antibodies used in this study were validated for specificity in the reported applications, and RRIDs were provided. On the following day, sections were incubated at room temperature for 2 h with appropriate fluorescent secondary antibodies: goat anti-chicken IgY Alexa Fluor 488 (Abcam Cat# ab150169, 1:500, RRID: AB_2636803), goat anti-rat IgG Alexa Fluor 647 (Cell Signalling Technology Cat# 4418, 1:500, RRID: AB_1904017), goat anti-guinea pig IgG Alexa Fluor 555 (Thermo Fisher Scientific Cat# A-21435, 1:500, RRID: AB_2535856), goat anti-chicken IgY Alexa Fluor 647 (Abcam Cat# ab150171, 1:500, RRID: AB_2921318), and goat anti-mouse IgG Alexa Fluor 488 (Cell Signalling Technology Cat# 4408, 1:500, RRID: AB_10694704), along with DAPI (Thermo Fisher Scientific Cat# D1306, 1:1000, RRID: AB_2629482) for nuclear staining. Images were acquired using a laser-scanning confocal microscope (LSM 900, Carl Zeiss, Germany). We quantified synaptic density with ImageJ software (RRID: SCR_003070), employing three-dimensional reconstruction and morphometric analyses through Imaris software (RRID: SCR_007370). This approach allowed for detailed visualisation and quantification of microglia-mediated synaptic engulfment, utilising Iba1-, CD68-, and synaptic protein-positive signals, in accordance with established methodologies.

### Microglia synaptic phagocytosis

The quantification of VGLUT1 or VGAT engulfment in microglia from mouse models was carried out using Imaris Reconstruction software (v.9.9, Bitplane), following established protocols.^72^^,^^73^ High-resolution confocal microscopy images of Iba1^+^ microglial cells, co-stained with CD68 and VGLUT1 or VGAT, provided the basis for these reconstructions. Image processing was performed in Imaris, which included background subtraction and the application of a Gaussian filter. Iba1^+^ microglia were rendered as surfaces with 0.1-μm smoothing; disconnected cellular processes were integrated with the main cell body to form a unified surface representation. A mask was applied to the CD68 channel to specifically extract the CD68 signal from within the microglial context for surface rendering, also utilising 0.1-μm smoothing. For the quantification of VGLUT1 or VGAT engulfment, masks were applied to the respective channels to highlight the signals localised within microglial lysosomes. The isolated VGLUT1 or VGAT signals were then surfaced and rendered (with 0.1-μm smoothing) to measure their volumetric contribution. The percentage of VGLUT1 or VGAT engulfment within the lysosomes was calculated using the formula: (volume of engulfed material within lysosome)/(volume of Iba1^+^ cell).

### Cell culture

The BV2 murine microglial cell line (RRID: CVCL_0182) used in this study was tested negative for mycoplasma contamination and was cultured in high-glucose DMEM (BasalMedia Cat# L110KJ, Shanghai) with 10% foetal bovine serum (FBS Cat# A5669701, Gibco, USA), under standard conditions (37 °C, 5% CO_2_, humidified atmosphere). For the isolation of primary microglia, neonatal mice aged 1–3 days were first sanitised with 75% ethanol before dissection. Cortical and hippocampal tissues were extracted and processed in ice-cold PBS. Following the removal of meninges, the brain tissues were minced into fine fragments and washed three times with cold PBS. These tissue pieces were then triturated using a pipette to create a uniform slurry. The resulting suspension was digested with 0.05% trypsin–EDTA (Gibco Cat# 25200072, USA) at 37 °C for 10 min, followed by further trituration to yield a single-cell suspension. The cells were subsequently seeded onto poly-d-lysine-coated culture plates and cultured in F-12/DMEM medium (Gibco Cat# C11330500BT, USA). After 3 days, the culture medium was replaced with F-12/DMEM supplemented with GM-CSF to enhance microglial proliferation. After a 12-day culture period, the flasks were agitated at 200 rpm for 2 h at 37 °C to detach the microglial cells. The supernatant containing the suspended microglia was collected and centrifuged at 1000 rpm for 10 min to pellet the cells. Finally, the cell pellet was resuspended in F-12/DMEM containing 10% FBS for use in subsequent experiments.

### Microglia phagocytosis assay

To evaluate the phagocytic effects of botulinum toxin type A (BoNT/A) on BV2 microglial cells or primary microglia activated with the C3aR agonist, we quantified the internalisation of latex beads as a measure of phagocytic activity.^72^ BV2 microglial cells or primary microglia were cultured on glass coverslips in 24-well plates. Prior to seeding, the coverslips were treated with 0.01 mg/mL poly-d-lysine (Sigma–Aldrich Cat# P0899, USA) at 37 °C for 2 h, followed by three washes with PBS to remove excess coating. After a 24-h treatment period with 100 nM C3a (ANNORON Cat# A118, Beijing) and BoNT/A, latex beads (Merck Cat# L1030, Germany) were pre-incubated in FBS diluted 1:5 at 37 °C for 1 h. This bead-FBS mixture was then diluted 1:2000 in the culture medium. Following the removal of the drug-containing medium, the bead-containing medium was added to the cells for an additional 4 h of incubation. The subsequent procedures adhered to the standard immunocytochemistry (ICC) protocol.

### Immunocytochemistry

Cells were subjected to three washes with pre-chilled PBS to eliminate any surface-bound latex beads, followed by fixation with 4% paraformaldehyde (PFA) at room temperature for 15 min. Blocking was performed using 5% normal goat serum in PBST for 1 h at room temperature. Primary antibodies were incubated overnight at 4 °C. The next day, cells were washed gently with PBS three times, each wash lasting 15 min. Secondary antibodies were applied for 2 h at room temperature. After the staining process, coverslips were mounted onto glass slides, and imaging was carried out using a Zeiss confocal microscope (model LSM800). Quantitative analysis was performed utilising ImageJ software (RRID: SCR_003070).

### Clinical sample collection and processing

This study involved the collection of 64 plasma samples from the Second Affiliated Hospital of Soochow University, equally divided between 32 male and 32 female participants. The samples included 9 male and 10 female healthy controls, 11 male and 10 female patients with PD, and 12 male and 12 female patients with PD who also had comorbid depression. All participants were self-identified as East Asian, specifically from the Yangtze River Delta region of China. Race and ethnicity were recorded by self-report at enrolment, consistent with local demographic and clinical research practices. The study was conducted in accordance with the guidelines of the Declaration of Helsinki and was approved by the Ethics Committee of the Second Affiliated Hospital of Soochow University (Approval No. JD-LK2023002-IR01). All participants provided informed consent. To preserve the samples for analysis, they were immediately snap-frozen in liquid nitrogen and subsequently stored at −80 °C. Protein concentration analysis was performed using the BCA Protein Assay Kit from Thermo Scientific (Cat# 23225).

### Animal tissue sample collection and protein extraction

To establish a model, three-month-old C57BL/6J mice underwent slow infusion of MPTP and probenecid. Following the modelling process, tissues from the hippocampus, prefrontal cortex, substantia nigra, and striatum were carefully collected. These samples were promptly snap-frozen in liquid nitrogen and stored at −80 °C to preserve their integrity for further analysis. Tissue homogenisation was accomplished using a tissue grinder, and protein extraction was performed with a lysis buffer consisting of 4% SDS and 50 mM HEPES (pH 8.0), supplemented with protease inhibitors (Sigma–Aldrich Cat# P8340-1 ML, USA). The extracted samples were centrifuged at 12,000 rpm for 10 min at 4 °C, after which the supernatant was collected for subsequent analysis. Protein concentration was assessed using the BCA Protein Assay Kit (Thermo Scientific, Cat# 23225).

### Sample preparation for mass spectrometry-based proteomics

For protein preparation, 1 mg of protein was added to a solution containing 10 mM TCEP and 40 mM IAA. This mixture was then incubated at 37 °C for 1 h to facilitate disulfide bond reduction and cysteine-SH alkylation. Following this reaction, we added five times the volume of ice-cold acetone to promote overnight protein precipitation. Once the protein was precipitated, it was dissolved in 8M urea with 50 mM HEPES buffer. We then introduced trypsin to achieve a protein-to-enzyme mass ratio of 50:1. The digestion was conducted at 37 °C with a shaking speed of 800 rpm for 16 h, allowing for thorough enzymatic activity overnight. After digestion, the resulting supernatant underwent purification and desalting using a C18 column from Waters (Cat# 2215-C18). Finally, the eluted peptides were vacuum-dried and stored at −80 °C for future applications.

### LC-MS (liquid chromatography mass spectrometry) analysis

LC-MS/MS analysis was performed using a Vanquish Neo UHPLC system coupled to an Orbitrap Astral mass spectrometer (Thermo Fisher Scientific). Peptides were separated under nanoLC conditions on a 15 cm × 100 μm analytical column using direct injection. The mobile phases consisted of Buffer A (0.1% formic acid in water) and Buffer B (80% acetonitrile containing 0.1% formic acid). The loading volume was 5 μL, and the autosampler temperature was maintained at 7 °C. The LC gradient was set as follows: 1% B at 0 min, 8% B at 0.5 min, 25% B at 6.6 min, 35% B at 10.4 min, 55% B at 10.9 min, and 99% B at 11.4 min, which was held until 13.0 min. The flow rate was 1.0 μL/min from 0 to 0.5 min, reduced to 0.7 μL/min from 0.6 to 11.4 min, and returned to 1.0 μL/min from 11.4 to 13.0 min. Mass spectrometric acquisition was performed in positive-ion data-independent acquisition (DIA) mode. Full MS scans were acquired in the Orbitrap analyser over an *m*/*z* range of 380–980 at a resolution of 240,000, with a scan time of 5 ms, a normalised AGC target of 500%, and an absolute AGC target of 5,000,000. DIA-MS/MS spectra were acquired in the Astral analyser over an *m*/*z* range of 150–2000, with a precursor mass range of 380–980, using 2 *m*/*z* isolation windows and 300 scan events. Fragmentation was performed using HCD with a normalised collision energy of 25%. The Astral scan time was 3 ms, with a normalised AGC target of 500% and an absolute AGC target of 50,000, and pre-accumulation was enabled. FAIMS was not installed, and ion source parameters were applied according to the Tune settings.

### Proteomics data processing and bioinformatics analysis

The data obtained from the experiment were systematically stored as raw files and subsequently analysed using DIA-NN software (version 1.8). This allowed for effective matching of the mass spectrometry results with the human or mouse FASTA protein database. To ensure accurate results, the first-order error tolerance was set at 15 ppm, while the second-order error tolerance was established at 20 ppm. For the protein digestion process, trypsin was applied with an enzymatic cleavage approach, and Carbamidomethylation on cysteine residues was designated as a fixed modification. Additionally, acetylation at the protein N-terminus, oxidation of methionine, and deamidation of asparagine and glutamine residues were incorporated as variable modifications.

All bioinformatics analyses were carried out utilising R (version 4.2.0) along with associated Bioconductor packages. The experimental data were first imported using the readxl package, followed by thorough data cleaning and calculations with the tidyverse package. To address any missing values, we employed K-Nearest Neighbours (KNN) imputation via the impute.knn function from the impute package, ensuring robust handling of randomly missing data points. Selection criteria for significant findings were based on an adjusted p-value using the Benjamini-Hochberg (BH) method for multiple testing correction, with a threshold of less than 0.05, coupled with an absolute log2 Fold Change exceeding 0.3785. For functional enrichment analysis, the clusterProfiler package was utilised to conduct Gene Ontology and KEGG enrichment analyses. The Gene Ontology (GO) analysis focused on biological processes (BP), cellular components (CC), and molecular functions (MF), while the KEGG pathway analysis explored enriched metabolic and signalling pathways. Enrichment significance was assessed using Fisher's exact test and the Benjamini-Hochberg method for multiple testing correction, with a threshold for adjusted p-value set at less than 0.05. Data visualisation efforts were carried out with the ggplot2 package, which facilitated the creation of informative volcano plots, heatmaps, and bar charts to effectively display differentially expressed proteins and the results of the enrichment analyses. All statistical analyses and visualisations were conducted within the RStudio (version 2023.03.0) environment, ensuring a comprehensive and clear presentation of the findings.

### Single-cell RNA library and sequencing

The hippocampus was isolated from 3-month-old mice treated with MPTP for 5 weeks and sequenced using the 10x Genomics platform.^74^ After dissociating the pellet with the Papain, the cell suspension was analysed with a TC20 Automated Cell Counter to assess cell size, concentration, and viability. Barcoded Single Cell 3’ Gel Beads were combined with cells, enzymes, and partitioning oil to create gel bead-in-emulsion (GEMs). After dissolving the gel beads, oligonucleotides and cell lysate produced 10x Barcoded cDNA, which was purified, amplified by PCR, and quantified for library construction. dsDNA was fragmented, treated with End Prep Enzyme Mix, and then subjected to size selection to recover approximately 300 bp of cDNA. Adaptors were added via T-A ligation, and PCR products were cleaned and validated before library preparation. The multiplexed libraries were sequenced on an Illumina NovaSeq instrument using a 2 x 150 bp paired-end configuration, with data analysis performed using NovaSeq Control Software and GAPipeline-1.6.

### Sequencing data processing and quality control

To generate FASTQ files and perform alignments, the Illumina basecall files (∗.bcl) were converted to FASTQ format using bcl2fastq. The sequencing files underwent filtering using Cutadapt (version 1.9.1) with dual indexing and a read length of 50bp. Subsequently, the filtered files were processed using the 10 × Cell Ranger 7.1.0 pipeline against a *Mus musculus* genome (GRCm38) reference to generate gene count data. In addition, we use CellBender (version 0.2.2) to remove ambient RNA molecules. The outputs were further analysed using R and R Studio (R version 4.3.2) with specific packages indicated below. Unless specified otherwise, all single-cell RNA sequencing (scRNA seq) was performed within the Seurat package v5.1.0. The decontaminated expression matrices were then further processed following the standard Seurat quality control pipeline. Briefly, low-quality cells with few genes expressed (<200), more genes expressed (>5000), and a proportion of mitochondrial genes >20% were removed.

### Dataset integration, clustering, and sub-clustering of cell types

To ensure the highest quality of our data, we initiated a comprehensive quality control pipeline, which included normalisation and variance stabilisation for each dataset. Following this crucial step, the datasets were meticulously combined and normalised using the “NormalizeData” function. To facilitate downstream analyses, we identified 2000 highly variable features through the “FindVariableFeatures” function. Subsequently, we scaled and centred the data for all genes utilising the “ScaleData” function. With our data effectively prepared, we proceeded with dimensionality reduction on all datasets, implementing principal component analysis (PCA) via the “RunPCA” command. This was followed by UMAP (Uniform Manifold Approximation and Projection) dimensionality reduction and Louvain clustering, set at a resolution of 0.5. This structured approach led us to the successful identification of 26 distinct cell clusters. To accurately determine cell identities, we employed supervised methods that included a thorough manual inspection of the marker gene list, assigning cell identities based on the expression of putative marker genes from the CellMarker 2.0 database. When a cluster is characterised by a single marker gene, it indicates that the selected gene is both selectively and robustly expressed by that cluster, making it a reliable marker. In pursuit of deeper insights, we conducted sub-clustering analyses on the clusters of interest. This involved sample-based splitting and the iterative application of normalisation, variance stabilisation, and integration using the same parameters as earlier. Through this refined pipeline, we successfully delineated microglia into 11 distinct clusters for further study.

### Differential gene expression analysis and enrichment analysis

The Seurat function ‘FindMarkers’ was employed to effectively perform differential expression analysis and identify variations among different samples within the clusters. For the comparison between samples, we established parameters of |log2fold change| > 0.25 and p-value-adj <0.05 as thresholds for identifying differentially expressed genes (DEGs). To gain a more thorough understanding of gene function, both up- and down-regulated genes were subjected to enrichment analysis. For this purpose, we utilised the clusterProfiler package (v4.10.0) in conjunction with the org.Mm.eg.db database (v3.18.0) to conduct Gene Ontology (GO) and Kyoto Encyclopedia of Genes and Genomes (KEGG) enrichment analyses. We focused on retaining only those enriched terms that had p-values of less than 0.05, ensuring a robust set of results for further exploration.

### Statistical analysis

Statistical analyses were conducted using GraphPad Prism version 8.3.0. Data normality was assessed using the Kolmogorov–Smirnov test for samples of size ≥5, while the Shapiro–Wilk test was appropriate for samples <5. For pairwise comparisons, an unpaired t-test was utilised when the data were normally distributed; otherwise, the Mann–Whitney test was employed. In the case of multiple group comparisons, one-way ANOVA was applied, followed by either Tukey's or Dunnett's post hoc tests for single-factor analyses. For two-factor analyses, two-way ANOVA with subsequent Tukey's post hoc testing was used. Results are expressed as mean ± SEM, with statistical significance set at ns (not significant), *∗P* < 0.05, *∗∗P* < 0.01, *∗∗∗P* < 0.001, *∗∗∗∗P* < 0.0001, ^#^*P* < 0.05, ^##^*P* < 0.01, ^###^*P* < 0.001, ^####^*P* < 0.0001.

### Role of funders

The funders of the study had no role in study design, data collection, data analysis, data interpretation, writing of the report, or the decision to submit the paper for publication.

## Results

### Proteomic profiling reveals sex-specific immune pathways in patients with Parkinson's disease comorbid with Depression

**Fig. 1:**
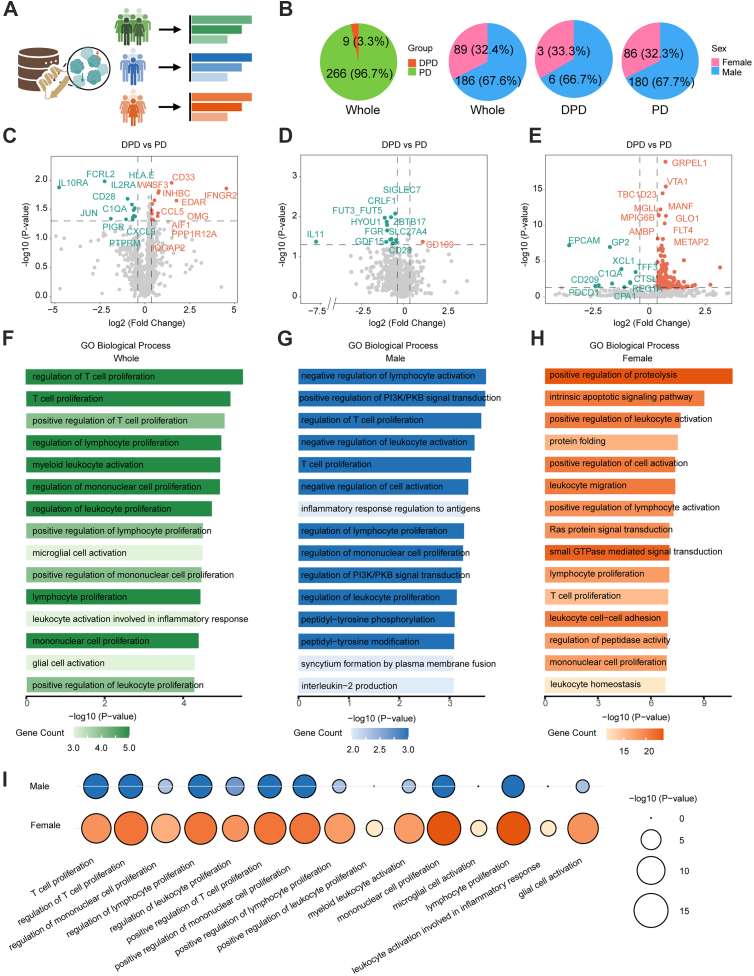
**Differential protein expression and enrichment analysis in male and female Parkinson's disease (PD) and Depression in Parkinson's disease (DPD) from the PPMI dataset. (A)** Overview of the study design. Baseline plasma protein data from PPMI were used (Olink NPX, log2 scale). After integrating phenotypes, *n* = 275 samples were included (DPD = 9, PD = 266; male = 186, female = 89). A two-group comparison (DPD vs PD) was performed at three levels—whole, male, and female. Significance was defined as |log2FC| > 0.3785 (≈1.3-fold) and *P* < 0.05. Significantly altered proteins were subjected to Gene Ontology Biological Process (GO-BP) enrichment to derive process profiles at each level. **(B)** Sample composition and grouping. The proportions of DPD and PD in the whole cohort are shown (DPD 3.3%, PD 96.7%), along with sex ratios for the whole/DPD/PD populations (whole: female 32.4%, male 67.6%; DPD: female 33.3%, male 66.7%; PD: female 32.3%, male 67.7%). Pie charts display counts and percentages. **(C**–**E)** Volcano plots. DPD vs PD for the whole **(C)**, male **(D)**, and female **(E)** cohorts. The x-axis is log2 fold change and the y-axis is −log10(P-value). Red/blue indicate significantly up-/down-regulated proteins; grey indicates non-significant proteins or |log2FC| < 0.3785. Vertical dashed lines mark the fold-change threshold (±0.3785), and the horizontal dashed line marks the significance threshold (*P* = 0.05). Representative significant proteins are labelled. **(F–H)** GO-BP enrichment analysis. The top 15 enriched processes are shown for the whole **(F)**, male **(G)**, and female **(H)** cohorts. The x-axis denotes −log10(P-value); colour intensity indicates the number of enriched genes (darker = more genes). The enrichment background is the set of detected, annotatable proteins in this study; terms are from GO Biological Process. **(I)** Enrichment analysis of the top 15 biological processes in the whole cohort across male and female populations, with colour legend and circle sizes consistent with **(G)** and **(H)**; larger circles indicate greater significance, and colour intensity represents the number of enriched genes (darker = more genes).

We first investigate the proteomic profiles of plasma from patients with Parkinson's disease (PD) with depressive symptoms (DPD) using data from the PPMI dataset (Fig. 1A). Differential expression analysis was conducted across 275 samples to compare protein profiles between patients with DPD and patients with PD-only, both overall and stratified by sex. A demographic analysis was summarised (Fig. 1B). Volcano plot analyses revealed differentially expressed proteins (DEPs) in DPD versus PD for the overall population (Fig. 1C), as well as for male (Fig. 1D) and female (Fig. 1E) cohorts. In the overall population, we identified 13 up-regulated proteins and 12 down-regulated proteins in DPD compared to PD (Fig. 1C). In the male cohort, DPD showed 1 up-regulated protein and 13 down-regulated proteins relative to PD (Fig. 1D). In contrast, the female cohort exhibited a significant increase, with DPD showing 236 up-regulated proteins and only 10 down-regulated proteins compared to PD (Fig. 1E). Functional enrichment analysis of these DEPs revealed significant immune-related pathways. In the overall population (Fig. 1F), Gene Ontology (GO) enrichment analysis identified prominent enrichment in biological processes associated with cell proliferation and immune cell activation. Specifically, there was a marked enrichment in pathways related to T cell proliferation, lymphocyte proliferation, and mononuclear cell proliferation, along with myeloid leucocyte activation and glial cell activation. The enrichment analysis for the male cohort shows significant enrichment in pathways related to cell signalling and immune responses. Key pathways include T cell proliferation and the regulation of lymphocyte activation, highlighting the role of specific signalling mechanisms in immune modulation in male patients with DPD (Fig. 1G). In contrast, distinct enrichment patterns were observed in the female population. Here, significant enrichment in the positive regulation of lymphocyte/leucocyte activation and small GTPase signalling pathways suggests that neuroinflammatory processes may be more positively modulated in females suffering from DPD (Fig. 1H). Finally, a comparative analysis of the significant shared biological pathways between male and female patients across the entire population was presented. Both sexes exhibit significant enrichment in immune-related pathways, particularly those involved in the regulation of T cell, mononuclear cell, lymphocyte, and leucocyte proliferation, as well as glial cell activation (Fig. 1I). In summary, GO enrichment analysis underscores significant involvement of immune and inflammatory responses in DPD. Moreover, while both sexes influence immune cell proliferation and microglial activation, the regulation of lymphocyte/leucocyte activation differs between males and females, highlighting sex-specific immune dysregulation in DPD pathogenesis.

### Plasma proteomic profiles in Parkinson's disease and comorbid depression across sexes

**Fig. 2:**
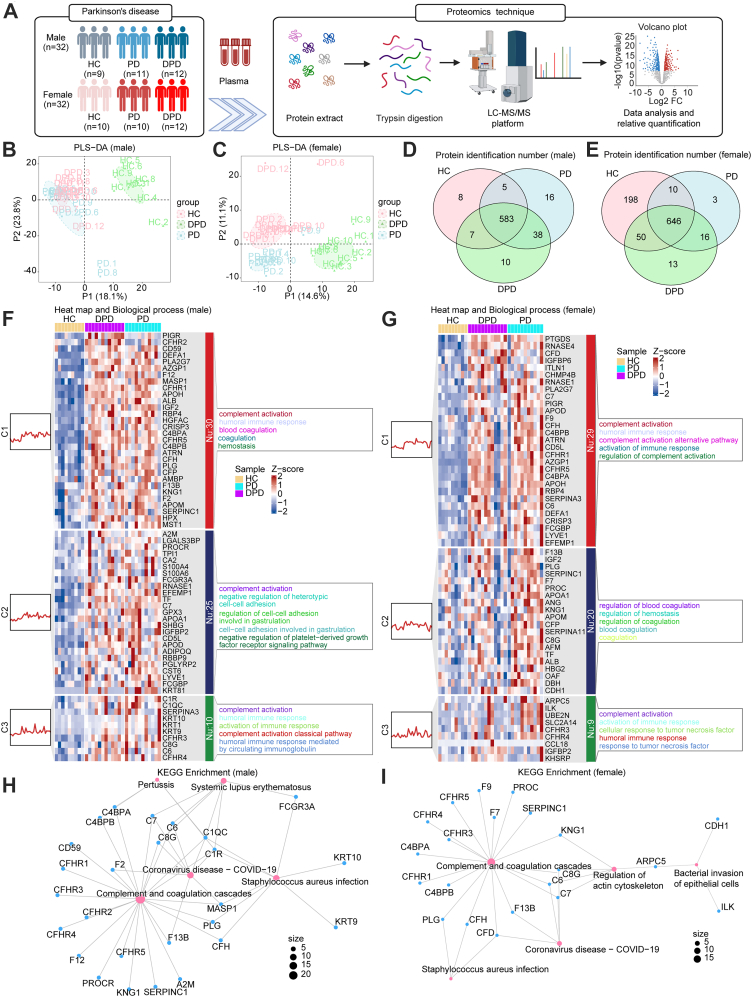
**Plasma proteomic profiles in Parkinson's disease and comorbid depression across sexes. (A)** Overview of the experimental design. Plasma samples from male (*n* = 32) and female (*n* = 32) subjects were analysed. The subjects were grouped as healthy controls (HC), Parkinson's disease (PD), and Depression in Parkinson's disease (DPD). Proteins were extracted from plasma, digested with trypsin, and analysed using an LC-MS/MS platform. Data were processed using various proteomics techniques. Created with Biorender.com. **(B–C)** Partial Least Squares-Discriminant Analysis (PLS-DA) plot for male **(B)** and female **(C)** subjects. The plot illustrates the separation of the HC, PD, and DPD groups, with each group distinguished by different colours and symbols. Variance explained by the first two principal components is shown as percentages on the axes. **(D**–**E)** Venn diagram illustrating the number of proteins identified across male **(D)** and female **(E)** groups. The Venn diagram effectively illustrates the differences in protein identification between the PD and DPD groups compared to the HC group. **(F**–**G)** Heatmap of biological processes in male **(F)** and female **(G)** subjects. Proteins were clustered based on their expression levels in the different groups (HC, PD, and DPD). The heatmap is coloured according to Z-scores. The biological processes are annotated on the right. **(H–I)** Kyoto Encyclopedia of Genes and Genomes (KEGG) enrichment analysis for male **(H)** and female **(I)** subjects. A network of enriched pathways is shown. The node size corresponds to the number of proteins associated with each pathway.

**Supplementary Figure 1:**
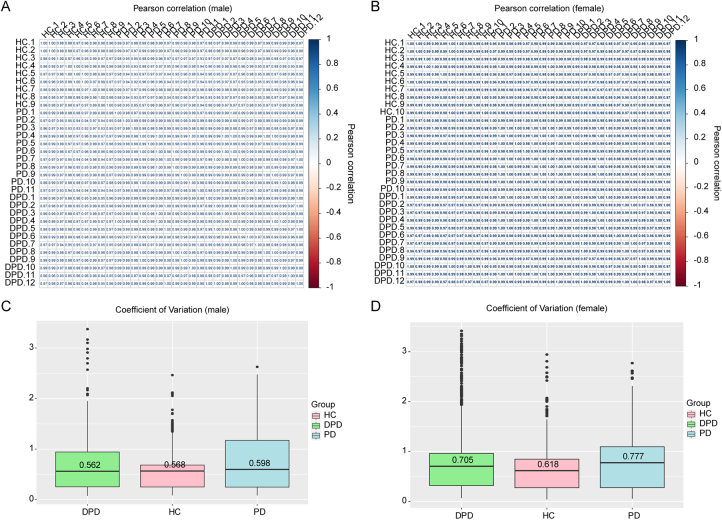
**Proteomic analysis of Parkinson's disease in male and female plasma samples. (A–B)** Pearson correlation matrices of male **(A)** and female **(B)** groups across various experimental conditions. Each cell represents the Pearson correlation coefficient between two conditions, ranging from −1 to 1, where values closer to 1 indicate a stronger positive correlation. **(C**–**D)** Coefficient of Variation (CV) analysis was conducted for male **(C)** and female **(D)** cohorts across various experimental conditions. The boxplots illustrate the distribution of CV values for the HC group, the DPD group, and the PD group. The numerical values within the boxplots indicate the median CV for each group.

To validate findings from the PPMI database, we conducted an independent cohort study that performed differential proteomic analysis of plasma from male and female participants with Parkinson's disease (PD), Depression in Parkinson's disease (DPD), and healthy controls (HC) (Fig. 2A). Supplementary Table S1 summarises the clinical characteristics of participants. Pearson correlation and coefficient of variation (CV) analysis indicated strong reproducibility across samples (Fig. S1A–D). The partial least squares discriminant analysis (PLS-DA) plots demonstrated a pronounced distinction among the DPD, PD, and HC cohorts for both male and female subjects, highlighting notable proteomic alterations (Fig. 2B and C). A summary of the proteins identified within each group is presented (Fig. 2D and E). These findings highlight the distinct proteomic signatures potentially involved in disease pathophysiology.

**Supplementary Figure 2:**
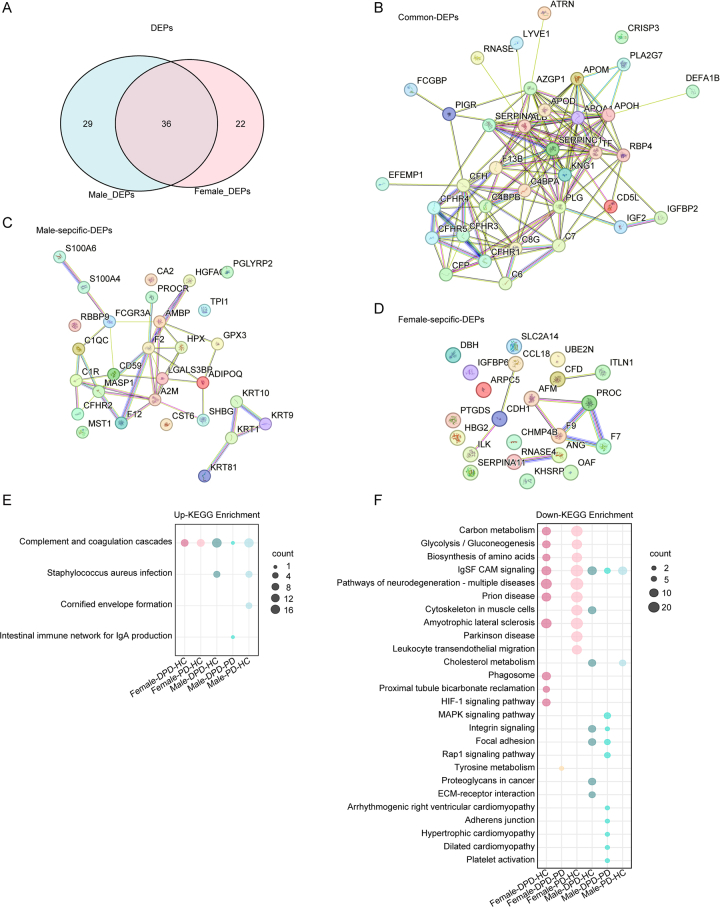
**Shared and sex-specific differentially expressed plasma proteins in Parkinson's disease and comorbid depression across sexes. (A)** Venn diagram illustrating the overlap of differentially expressed proteins (DEPs) identified under the criteria: nominal P value < 0.05 and fold change (FC) ≥ 1.1. In total, 36 DEPs were shared between male and female groups, with 29 DEPs unique to males and 22 DEPs specific to females. **(B**–**D)** Protein–protein interaction (PPI) networks of shared DEPs **(B)**, male-specific DEPs **(C)**, and female-specific DEPs **(D)**. Nodes represent proteins and edges represent predicted interactions between proteins. **(E**–**F)** KEGG pathway enrichment analyses of upregulated **(E)** and downregulated **(F)** DEPs in male and female subjects. Each dot indicates significantly enriched KEGG pathways for each comparison (PD vs HC, DPD vs HC, and DPD vs PD).

Protein identification was next performed across three study cohorts: HC, PD, and DPD. Pairwise comparative analyses were subsequently conducted to screen for differentially expressed proteins (DEPs) via unpaired two-tailed Student's t-tests between PD versus HC and DPD versus HC, respectively. Hierarchical clustering analysis was then performed separately for male and female subgroups based on the normalised expression levels of the filtered DEPs, resulting in the stratification of these proteins into three distinct clusters: C1, C2, and C3, respectively. We performed Gene Ontology (GO) biological process enrichment analysis on DEPs within distinct protein clusters separately for males and females (Fig. 2F and G). To further characterise these disease-associated DEPs across sexes, we conducted a Venn analysis. A total of 36 shared DEPs were identified between males and females, while 29 DEPs were specifically altered in males and 22 DEPs were unique to females (Fig. S2A–D). The shared DEPs in both male and female PD and DPD were highly enriched in complement activation, coagulation-fibrinolysis imbalance, lipid metabolic disturbance, mucosal immunity, and gut–brain axis regulation (Fig. 2F and G, Fig. S2B). CFH, CFHR1/3/4/5, C4BPA/B, CFP, and C6/C7/C8G covered the alternative, classical, and terminal MAC assembly pathways, suggesting that excessive complement activation represents a core trans-sexual pathological mechanism. PLG, F13B, KNG1, and SERPINC1 jointly drove dysregulated coagulation and fibrinolysis. APOA1, APOD, APOM, RBP4, AZGP1, PLA2G7, and CD5L contributed to impaired lipid transport and antioxidative function. PIGR, DEFA1B, FCGBP, and LYVE1 indicated disrupted mucosal immunity and intestinal barrier function, supporting the involvement of the gut–brain axis in PD and DPD. Collectively, these shared proteins demonstrate that complement–coagulation axis dysfunction, immune-metabolic disorders, and gut–brain axis disturbance represent conserved core pathological mechanisms across sexes in PD and DPD.

Beyond these conserved pathological signatures, distinct sex-specific proteomic profiles were identified in patients with PD and DPD, with unique functional perturbations observed in the male cohort (Fig. 2F, Fig. S2C). Male-specific DEPs in PD and DPD were also associated with classical complement pathway activation, coagulation cascades, cytoskeletal remodelling, protease inhibition, and regulation of oxidative stress. Among them, C1R and C1QC indicated aberrant activation of the classical complement pathway, while F2, F12, A2M, and PROCR contributed to disrupted coagulation-anticoagulant homoeostasis. S100A4, S100A6, and multiple keratins (KRT1, KRT9, KRT10, and KRT81) reflected enhanced cytoskeletal reorganisation and cellular stress. FCGR3, LGALS3BP, and PGLYRP2 were involved in immune recognition and inflammatory regulation, and GPX3, AMBP, TF, and HPX participated in antioxidative responses and metabolic balance, collectively defining a male-specific pathological phenotype characterised by disturbance of the complement–coagulation axis, cytoskeletal abnormalities, and neuroinflammation.

In contrast, female patients exhibited a wholly distinct set of dysregulated proteins linked to immune, metabolic, and neurotransmitter-related pathways, further underscoring marked sexual dimorphism in the molecular pathogenesis of PD and DPD (Fig. 2G, Fig. S2D). In females, uniquely elevated proteins in PD and DPD were predominantly linked to alternative complement initiation, immune chemotaxis, neurotransmitter metabolism, angiogenesis, and cell adhesion. CFD, a key initiator of the alternative complement pathway, together with CCL18, promoted macrophage recruitment and neuroinflammation. DBH, a critical enzyme in norepinephrine synthesis, was closely associated with depressive symptoms and sympathetic dysfunction. PROC, F7, F9, and ANG modulated coagulation and vascular homoeostasis, whereas ARPC5, ILK, and CDH1 mediated cytoskeleton and cell–cell junction remodelling. SLC2A14, IGFBP6, and PTGDS were involved in glucose metabolism, growth factor signalling, and sleep-mood regulation, highlighting a female-specific pathological profile characterised by immune chemotaxis, neurotransmitter imbalance, and metabolic-emotional crosstalk.

Following the clustering and functional annotation workflow, Kyoto Encyclopedia of Genes and Genomes (KEGG) enrichment analysis of sex-stratified differentially expressed proteins revealed that complement and coagulation cascades, *Staphylococcus aureus* infection, and COVID-19 pathways were significantly dysregulated in both males and females, indicating immune and coagulation perturbations in PD and DPD (Fig. 2H and I).

### Conserved and sex-specific functional enrichment signatures in PD with comorbid depression

**Fig. 3:**
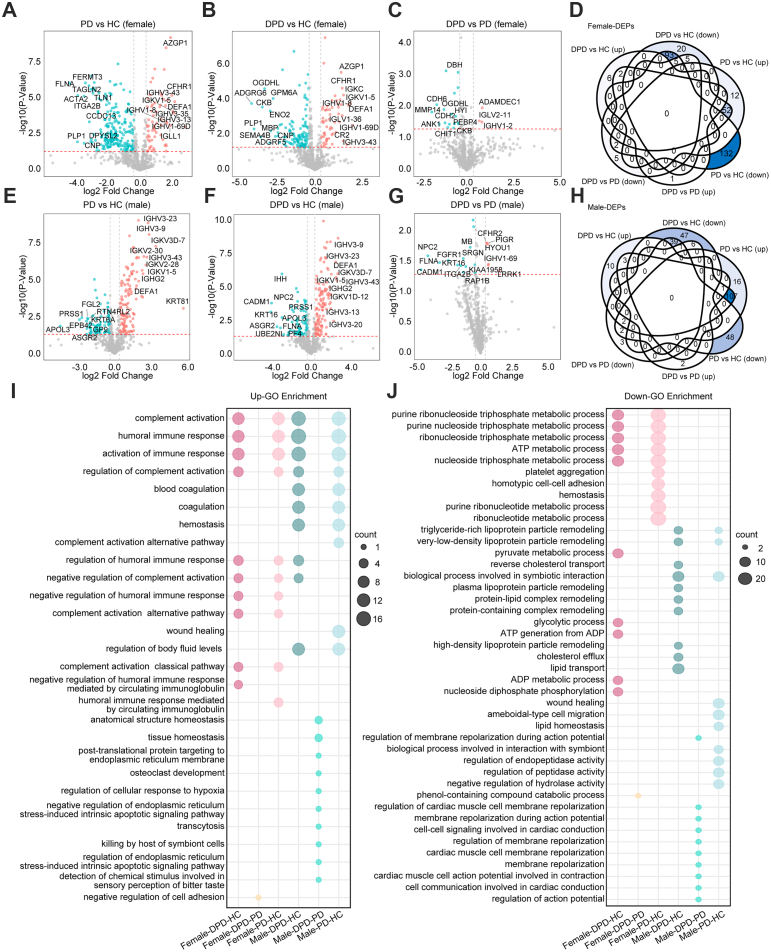
**Conserved and sex-specific functional enrichment signatures in PD with comorbid depression. (A**–**C)** Volcano plots provide a comprehensive overview of differentially expressed proteins (DEPs) for female subjects. Panel **(A)** examines the comparison between the PD and HC groups, while Panel **(B)** highlights the differences between the DPD and HC. Panel **(C)** addresses the comparison of DPD versus PD. Each plot effectively presents the log2 fold change on the x-axis alongside the -log10 p-value on the y-axis. To enhance clarity, significant proteins are represented with coloured dots—red for those that are up-regulated and blue for those that are down-regulated—while non-significant proteins are displayed in grey. The dashed line represents the threshold for statistical significance. **(D)** Venn diagram illustrates the overlap of DEPs in female subjects across three comparisons: PD versus HC, DPD versus HC, and DPD versus PD. Each group is distinctly colour-coded, with the intersections highlighting shared DEPs. Female-specific DEPs are indicated with blue shading, while proteins common across the comparisons are represented in the overlapping regions. **(E**–**G)** Volcano plots illustrating DEPs for male subjects are presented. Panel **(E)** compares PD against the HC, panel **(F)** compares DPD versus HC, and panel **(G)** illustrates DPD compared to PD. Proteins exhibiting significant expression changes are highlighted, with the dashed line representing the significance threshold. **(H)** Venn diagram illustrates the distribution of DEPs in male subjects across three comparisons: PD versus HC, DPD versus HC, and DPD versus PD. The DEPs unique to male subjects are highlighted in blue, while the overlapping DEPs found across the comparisons are situated within the intersection of the circles. **(I**–**J)** GO enrichment analysis for upregulated **(I)** and downregulated **(J)** differentially expressed proteins in female and male subjects. The dot indicates significantly enriched biological processes for each comparison (DPD vs HC, DPD vs PD, and PD vs HC).

To further investigate the dysregulated DEPs identified in PD, DPD, and HC groups, stratified by sex, volcano plots showed the top 10 up- and down-regulated proteins for both male and female subjects (Fig. 3A–C, Fig. 3E–G). Venn diagrams revealed both overlapping and distinct DEPs between PD and DPD groups in both females and males, indicating shared and divergent pathological mechanisms (Fig. 3D and H). To elucidate the functional roles of the identified differentially expressed proteins (DEPs), Gene Ontology (GO) biological process (BP) and Kyoto Encyclopedia of Genes and Genomes (KEGG) pathway enrichment analyses were performed (Fig. 3I and J, Fig. S2E and F), with stratification by sex and pairwise comparison group. For GO biological process enrichment in the PD versus HC and DPD versus HC comparisons, upregulated DEPs in both male and female cohorts were significantly enriched in the core functional terms of complement activation and activation of immune response, indicating conserved innate immune dysregulation across sexes (Fig. 3I). In contrast, downregulated DEPs showed marked sexual dimorphism, with minimal overlap in enriched terms between males and females. Female downregulated DEPs were predominantly enriched in nucleoside triphosphate metabolic process, while male downregulated DEPs were mainly associated with lipoprotein particle remodelling (Fig. 3J). In the direct DPD versus PD comparison, significant GO BP enrichment was observed in male DEPs. Upregulated male DEPs in this comparison were linked to key processes, including transcytosis, anatomical structure and tissue homoeostasis, regulation of cellular response to hypoxia, and modulation of endoplasmic reticulum stress-induced intrinsic apoptotic signalling (Fig. 3I). Conversely, downregulated male DEPs were overwhelmingly enriched in pathways governing membrane repolarisation during action potential (Fig. 3J). For female DEPs, only limited functional enrichment was identified, with upregulated DEPs associated with negative regulation of cell adhesion and downregulated DEPs linked to phenol-containing compound catabolic processes (Fig. 3I and J).

Consistent with the GO biological process findings, KEGG pathway enrichment analysis corroborated strong conservation and distinct sexual divergence in pathway dysregulation (Fig. S2E and F). In both PD versus HC and DPD versus HC comparisons, upregulated DEPs from both sexes were consistently enriched in the complement and coagulation cascades, reinforcing the central role of complement immune dysfunction in PD and DPD pathogenesis (Fig. S2E). For downregulated DEPs, only IgSF CAM signalling was shared between males and females, with distinct sex-specific pathway differences. Female downregulated DEPs were significantly enriched in core metabolic and neurodegenerative pathways, including carbon metabolism, glycolysis/gluconeogenesis, biosynthesis of amino acids, and multiple disease-associated neurodegeneration pathways, while male downregulated DEPs were selectively enriched in cholesterol metabolism (Fig. S2F). Parallel to the GO results, KEGG enrichment in the DPD versus PD comparison was restricted to male DEPs, with no significant pathway enrichment observed in females. Upregulated male DEPs were specifically enriched in the intestinal immune network for IgA production (Fig. S2E), highlighting gut-associated immune dysregulation as a male-specific feature of depressive comorbidity in PD. Downregulated male DEPs were enriched in platelet activation, and multiple cardiomyopathy-related pathways, alongside adherens junction regulation. In female DPD versus PD comparisons, the only significant KEGG enrichment was detected for downregulated DEPs, which were associated with tyrosine metabolism (Fig. S2F). Collectively, GO biological process and KEGG pathway enrichment analyses uncovered both conserved and sex-divergent functional perturbations underlying Parkinson's disease (PD) and PD with comorbid depression (DPD), with striking sexual dimorphism evident in the direct DPD versus PD comparison. These findings highlight profound sexual dimorphism in the molecular pathophysiology of depressive comorbidity in PD, with common and distinct functional alterations driving disease heterogeneity between male and female patients.

### Complement-mediated inflammation and region-specific protein dysregulation in the MPTP-induced Parkinsonian mouse model

**Fig. 4:**
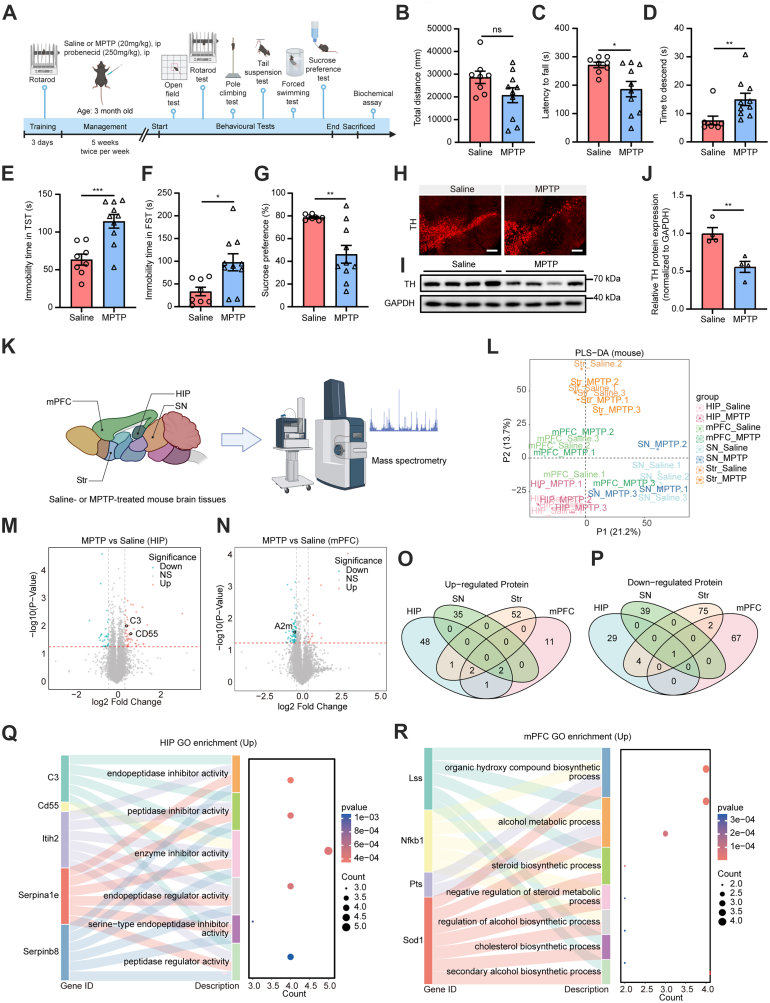
**Proteome profiling across brain regions of the MPTP/probenecid-induced Parkinsonian mouse model. (A)** Experimental timeline: Mice received either saline or MPTP (20 mg/kg, i.p.), followed 1 h later by probenecid (250 mg/kg, i.p.), twice a week for five weeks. Behavioural tests included the open field test, rotarod test, pole climbing test, tail suspension test (TST), forced swimming test (FST), and sucrose preference test (SPT). Mice were sacrificed for biochemical assays and protein analysis. Created with Biorender.com. **(B**–**G)** Behavioural results: total distance travelled in the open field test **(B)**, latency to fall in the rotarod test **(C)**, time to descend in the pole climbing test **(D)**, immobility time in TST **(E)** and FST **(F)**, and sucrose preference percentage in the SPT **(G)**. *n* = 8 saline, *n* = 10 MPTP mice. **(H**–**J)** Immunostaining and immunoblotting showed TH expression in the SNpc, with quantification normalised to GAPDH (*n* = 4/group). Scale bar = 100 μm. **(K)** Workflow used for sample processing and analysis. Brain regions (mPFC, HIP, Str, SN) were dissected from mice treated with either Saline or MPTP. Following labelling, all samples were pooled, fractionated using basic reversed-phase chromatography, and analysed by LC-MS/MS. Created with Biorender.com. **(L)** PLS-DA plot for protein expression variance in different brain regions (mPFC, HIP, SN, Str) of saline and MPTP-treated mice. **(M**–**N)** Volcano plots for differential protein expression in the HIP **(M)** and mPFC **(N)** regions comparing MPTP to saline treatments. **(O–P)** Venn diagrams showing the overlap of upregulated **(O)** and downregulated **(P)** proteins across the brain regions (HIP, SN, Str, mPFC) in MPTP/probenecid-treated mice compared to saline controls. **(Q**–**R)** GO enrichment analysis of DEPs of upregulated DEPs from HIP **(Q)** and mPFC **(R)** between saline- and MPTP-treated groups. All data are presented as the mean ± SEM. Significance was assessed by Student's t-test. ∗*P* < 0.05, ∗∗*P* < 0.01, ∗∗∗*P* < 0.001, versus Saline group.

Given the convergent role of complement-mediated inflammation in both female and male patients with DPD, and considering avoiding potential confounding effects of oestrogen, we primarily used male mice to establish a chronic MPTP/probenecid-induced Parkinsonian model and investigate the involvement of the complement cascade in PD with comorbid depression. Motor and depressive-like behaviours were evaluated through a comprehensive battery of tests (Fig. 4A). Compared to saline-treated controls, MPTP-treated mice showed significant motor deficits, including reduced total distance travelled in the open field test, decreased latency to fall in the rotarod test, and prolonged descent time in the pole test (Fig. 4B–D). Depressive-like phenotypes were similarly evident, with MPTP administration increasing immobility time in both the tail suspension test (TST) and forced swimming test (FST), and sucrose preference test (SPT) revealed marked anhedonia (Fig. 4E–G). Immunofluorescence and Western blot analyses confirmed dopaminergic degeneration, demonstrating significantly reduced tyrosine hydroxylase (TH) expression in the substantia nigra (SN) (Fig. 4H–J). Collectively, these results establish that chronic MPTP exposure recapitulates core features of PD with depression, including motor dysfunction and behavioural despair, providing a validated model for mechanistic studies.

**Supplementary Figure 3:**
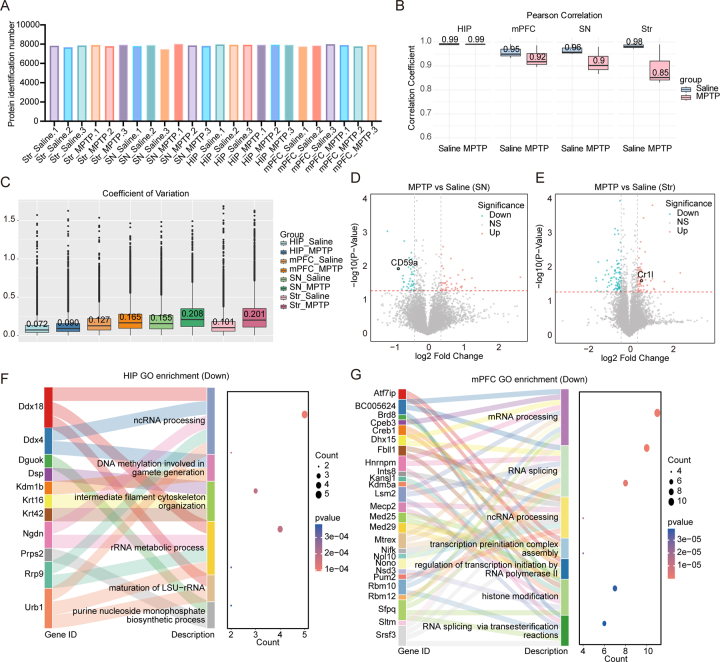
**Differential protein expression and pathway analysis in an MPTP Parkinsonian mouse model. (A)** Protein identification numbers for various brain regions (Str, SN, HIP, and mPFC) in saline and MPTP/probenecid-treated mice. The graph illustrates the proteomic profiling of various regions, contrasting the number of proteins identified in saline versus MPTP-treated groups. **(B)** Pearson correlation analysis of protein expression across brain regions in saline- and MPTP-treated groups. The correlation coefficients for HIP, mPFC, SN and Str are shown, indicating the degree of similarity between protein profiles in different regions. **(C)** Coefficient of variation for protein expression in different regions and groups. Values for each brain region and treatment group are shown, with statistical values (median CV) indicated on the bars. **(D**–**E)** Volcano plots of differential protein expression for the SN **(D)** and Str **(E)** regions comparing MPTP to saline-treated mice. Proteins with significant changes in expression are shown in red (up-regulated) and blue (down-regulated), while non-significant proteins are shown in grey. **(F**–**G)** Gene Ontology (GO) enrichment analysis for down-regulated proteins in the HIP **(F)** and mPFC **(G)** regions. The Sankey diagrams on the left illustrate the relationships between representative differentially expressed genes (Gene ID) and enriched GO terms (Description). The dot plots on the right summarise the enriched GO terms; dot size indicates gene count and colour indicates the enrichment p value.

Next, we performed mass spectrometry to identify the differentially expressed proteins (DEPs) across emotion- and motor-related brain regions from chronic MPTP-treated mice, including the medial prefrontal cortex (mPFC), hippocampus (HIP), striatum (Str), and substantia nigra (SN) (Fig. 4K). The same brain regions from different animals clustered together in PLS-DA and coefficient of variation, indicating consistency of tissue dissection (Fig. 4L and Fig. S3A–C). Volcano plots revealed region-specific protein alterations (Fig. 4M and N, and Fig. S3D and E). In the hippocampus, DEPs included significantly upregulated complement protein C3 and CD55 in MPTP-treated mice, suggesting their potential role in the pathophysiology of this model (Fig. 4M). Venn diagram analysis of upregulated proteins showed no overlap across emotion-related (HIP, mPFC) and motor-related (SN, Str) regions (Fig. 4O). However, within the HIP and mPFC, five proteins were co-upregulated: mammalian ependymin-related protein 1 (EPDR1), superoxide dismutase (SODC), 6-pyruvoyl tetrahydrobiopterin synthase (PTPS), immunoglobulin kappa constant (IGKC), and immunoglobulin heavy constant gamma 2B (IGG2B) (Fig. 4O). Notably, PTPS deficiency impairs synthesis of tetrahydropterin, a cofactor essential for monoamine neurotransmitter production, potentially linking it to emotional dysregulation.^75^ In contrast, the SN and Str showed no overlapping up-regulated proteins. On the other side, insulin-degrading enzyme (IDE) was consistently reduced across both emotion- and motor-related brain regions (Fig. 4P).

GO enrichment analysis of upregulated proteins in the HIP revealed significant enrichment in peptidase inhibitor activity, involving C3, Cd55, Itih2, Serpina1e, and Serpinb8 (Fig. 4Q). In the mPFC, upregulated proteins, such as Lss, Nfkb1, Pts, and Sod1, were notably enriched in alcohol metabolic and steroid biosynthetic processes (Fig. 4R). In contrast, downregulated proteins in both the HIP and mPFC regions were associated with RNA processing (Fig. S3F and G). Overall, our proteomic profiling of MPTP-treated mice reveals distinct, region-specific patterns of protein dysregulation across emotion- and motor-related brain areas, underscoring the distinct molecular alterations associated with specialised brain functions.

### Complement-mediated microglial synaptic pruning in the hippocampus of the MPTP Parkinsonian mouse model

**Fig. 5:**
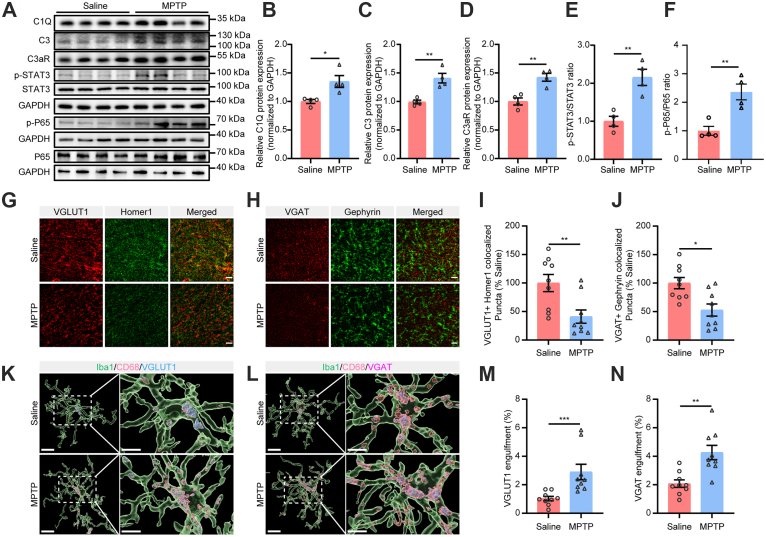
**Complement-mediated microglial synaptic phagocytosis in the hippocampus of the MPTP-induced Parkinsonian mouse model. (A)** Representative Western blot analyses showcasing the expression levels of complement proteins within the C3–C3aR signalling pathway and associated downstream effectors. **(B–F)** Statistical analysis of protein expression levels for C1Q **(B)**, C3 **(C)**, C3aR **(D)**, phosphorylated-/total-STAT3 **(E)**, and phosphorylated-/total-P65 **(F)**. *n* = 4 mice. **(G**–**H)** Representative images of excitatory synapses in the hippocampal CA1 region show colocalisation of VGLUT1 (red) and Homer1 (green) **(G)**, while inhibitory synapses are indicated by VGAT (red) and Gephyrin (green) **(H)**. Scale bar = 10 μm. **(I**–**J)** Quantitative analysis of colocalised VGlut1^+^Homer1^+^ intensity **(I)** and colocalised VGAT^+^Gephyrin^+^ intensity **(J)** in the hippocampal CA1 region. *n* = 9 images from 3 mice (3 images per mouse). **(K**–**L)** Representative Imaris images to visualise the microglial marker Iba1 (green), the lysosomal marker CD68 (pink), and the excitatory presynaptic marker VGLUT1 (blue) **(K)** or the inhibitory presynaptic marker VGAT (purple) **(L)** in the hippocampal region. Scale bar: left image, 10 μm; zoomed-in image, 5 μm. **(M**–**N)** Statistical analysis of the percentage of synaptic VGLUT1^+^ volume **(M)** or VGAT^+^ volume **(N)** in relation to the CD68^+^Iba1^+^ microglial volume. *n* = 9 images from 3 mice (3 images per mouse). All data are presented as the mean ± SEM. Significance was assessed by Student's t-test. ∗*P* < 0.05, ∗∗*P* < 0.01, ∗∗∗*P* < 0.001, versus Saline group.

**Supplementary Figure 4:**
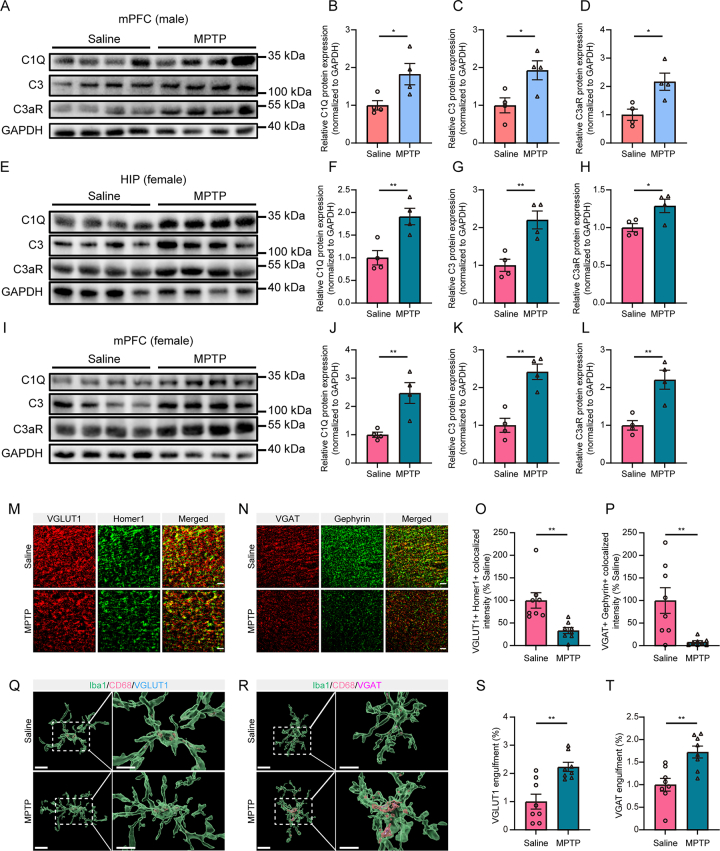
**Complement-mediated microglial synaptic phagocytosis in the MPTP/probenecid-induced Parkinsonian mouse model. (A)** Representative Western blot analyses of male mPFC lysates showcasing the expression levels of complement proteins C1Q, C3, and C3aR. **(B**–**D)** Statistical analysis of protein expression levels for C1Q **(B)**, C3 **(C)**, and C3aR **(D)**, *n* = 4 mice. **(E)** Representative Western blot analyses of female hippocampal lysates showing the expression levels of complement proteins C1Q, C3, and C3aR. **(F–H)** Statistical analysis of protein expression levels for C1Q **(F)**, C3 **(G)**, and C3aR **(H)**, *n* = 4 mice. **(I)** Representative Western blot analyses of female mPFC lysates showcasing the expression levels of complement proteins C1Q, C3, and C3aR. **(J**–**L)** Statistical analysis of protein expression levels for C1Q **(J)**, C3 **(K)**, and C3aR **(L)**, *n* = 4 mice. **(M**–**N)** Representative images of excitatory synapses in the female hippocampal CA1 region show colocalisation of VGLUT1 (red) and Homer1 (green) **(M)**, while inhibitory synapses are indicated by VGAT (red) and Gephyrin (green) **(N)**. Scale bar = 10 μm. **(O–P)** Quantitative analysis of colocalised VGlut1^+^Homer1^+^ intensity **(O)** and colocalised VGAT^+^Gephyrin^+^ intensity **(P)** in the hippocampal CA1 region. *n* = 8 images from 4 mice (2 images per mouse). **(Q**–**R)** Representative Imaris images to visualise the microglial marker Iba1 (green), the lysosomal marker CD68 (pink), and the excitatory presynaptic marker VGLUT1 (blue) **(Q)** or the inhibitory presynaptic marker VGAT (purple) **(R)** in the female hippocampal region. Scale bar: left image, 10 μm; zoomed-in image, 5 μm. **(S**–**T)** Statistical analysis of the percentage of synaptic VGLUT1^+^ volume **(S)** or VGAT^+^ volume **(T)** in relation to the CD68^+^Iba1^+^ microglial volume. *n* = 8 images from 4 mice (2 images per mouse). All data are presented as the mean ± SEM. Significance was assessed by Student's t-test. ∗*P* < 0.05, ∗∗*P* < 0.01.

Given the established association of complement activation with both clinical Parkinson's disease and experimental Parkinsonian models, we investigated complement protein expression within the male and female hippocampus and mPFC. Western blot analysis revealed significantly elevated levels of complement components C1Q, C3, and its receptor C3aR in hippocampal and mPFC lysates from MPTP-treated male and female mice compared to saline controls (Fig. 5A–D, Fig. S4A–L). Furthermore, aligning with previously identified C3aR signalling targets,^76^^,^^77^ we observed concomitant upregulation of phospho-/total-STAT3 and phospho-/total-P65 in the MPTP group (Fig. 5A, E, and F). The notable increase in complement components C1Q, C3, and C3aR in the hippocampus and mPFC suggests a potential enhancement of microglial activation for synaptic pruning in the MPTP model. Accordingly, we detected a significant reduction in densities of both excitatory (VGLUT1/Homer1) and inhibitory (VGAT/Gephyrin) synapses in the hippocampus of MPTP-treated male and female mice (Fig. 5G–J, Fig. S4M−P). Using 3D Imaris reconstruction of microglia stained for Iba1, CD68, and either VGLUT1 or VGAT, we further observed a marked increase in the volume of VGLUT1^+^ or VGAT^+^ synaptic material within CD68^+^ lysosomes of hippocampal microglia from MPTP-treated male and female mice (Fig. 5K–N, Fig. S4Q–T), indicating elevated microglial phagocytosis of synapses in both male and female mice.

**Fig. 6:**
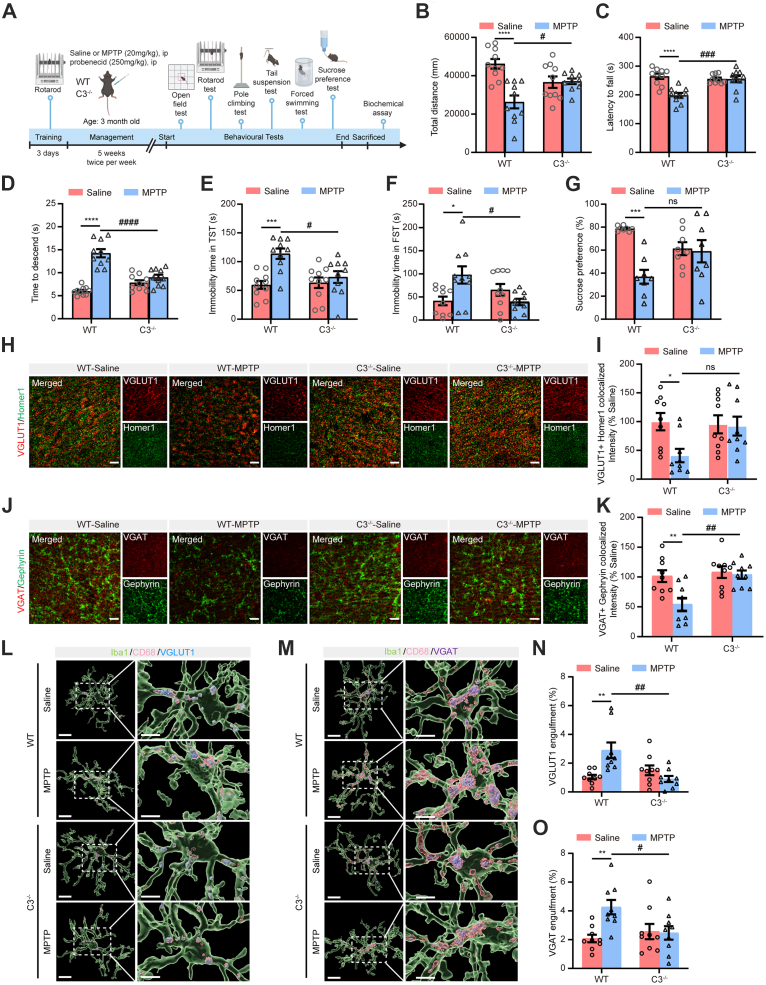
**Complement *C3* deficiency ameliorated abnormal behaviours, preserved synaptic integrity, and suppressed microglial synaptic phagocytosis in the MPTP/probenecid-induced Parkinsonian mouse model. (A)** Timeline for the MPTP/probenecid-induced PD model in *C3*-deficient mice. C57BL/6J and *C3* knockout mice received saline or MPTP (20 mg/kg, i.p.), followed 1 h later by probenecid (250 mg/kg, i.p.), twice weekly for five weeks. Behavioural tests, including open field, rotarod, pole climbing, TST, FST, and SPT, were conducted at various time points. Mice were sacrificed at the experiment's conclusion for biochemical assays. Created with Biorender.com. **(B)** The total distance travelled in the open field test. *n* = 10 mice/group. **(C)** The latency to fall in the rotarod test. *n* = 10 mice/group. **(D)** The time to descend in the pole climbing test. *n* = 10 mice/group. **(E)** Immobility time in the TST. *n* = 10 mice/group. **(F)** Immobility time in the FST. *n* = 10 mice/group. **(G)** Sucrose preference percentage in the SPT. *n* = 8 mice/group. **(H)** Representative images of excitatory synapses in the hippocampal CA1 region show colocalisation of VGLUT1 (red) and Homer1 (green). Scale bar = 10 μm. **(I)** Quantitative analysis of colocalised VGlut1^+^Homer1^+^ intensity in the hippocampal CA1 region. *n* = 8–9 images from 3 mice. **(J)** Representative images of inhibitory synapses are indicated by VGAT (red) and Gephyrin (green). Scale bar = 10 μm. **(K)** Quantitative analysis of colocalised VGAT^+^Gephyrin^+^ intensity in the hippocampal CA1 region. *n* = 8–9 images from 3 mice. **(L**–**M)** Representative Imaris images to visualise the microglial marker Iba1 (green), the lysosomal marker CD68 (pink), and the excitatory presynaptic marker VGLUT1 (blue) **(L)** or the inhibitory presynaptic marker VGAT (purple) **(M)** in the hippocampal region. Scale bar: left image, 10 μm; zoomed-in image, 5 μm. **(N–O)** Statistical analysis of the percentage of synaptic VGLUT1^+^ volume **(N)** or VGAT^+^ volume **(O)** in relation to the CD68^+^Iba1^+^ microglial volume. *n* = 9 images from 3 mice. All data are presented as the mean ± SEM. Significance was assessed by two-way ANOVA with Tukey's post hoc test. versus WT + Saline group: ∗*P* < 0.05, ∗∗*P* < 0.01, ∗∗∗*P* < 0.001, ∗∗∗∗*P* < 0.0001. versus WT + MPTP group: ^#^*P* < 0.05, ^##^*P* < 0.01, ^####^*P* < 0.0001.

To explore the role of complement C3 in microglia-mediated synaptic pruning associated with depressive-like symptoms in a Parkinsonian mouse model, we employed MPTP-treated *C3*-knockout (*C3*^*−/−*^) mice. We investigated the effects of *C3* loss-of-function on motor and depressive-like behaviours related to PD (Fig. 6A). Behavioural tests showed that *C3* deletion significantly improved MPTP-induced motor deficits, including reduced total distance travelled in the open field test, decreased latency to fall in the rotarod test, and increased descending time in the pole test (Fig. 6B–D). Furthermore, *C3* knockout rescued depressive-like behaviours, as indicated by shortened immobility time in the TST and FST, and increased sucrose preference (Fig. 6E–G). Moreover, regarding synapse loss in the hippocampus of MPTP-treated mice, *C3* deficiency prevented the loss of both excitatory and inhibitory synapses in the hippocampus (Fig. 6H–K). Importantly, this deletion also led to a reduction in microglial synapse engulfment compared to control mice (Fig. 6L–O). Together, these results demonstrate that complement C3 is a critical mediator of microglia-dependent synaptic loss and depressive-like symptoms in this Parkinsonian mouse model.

### BoNT/A targeting complement C3–C3aR pathway modulated microglial phagocytic activity in a rodent model of Parkinson's disease induced by MPTP

**Fig. 7:**
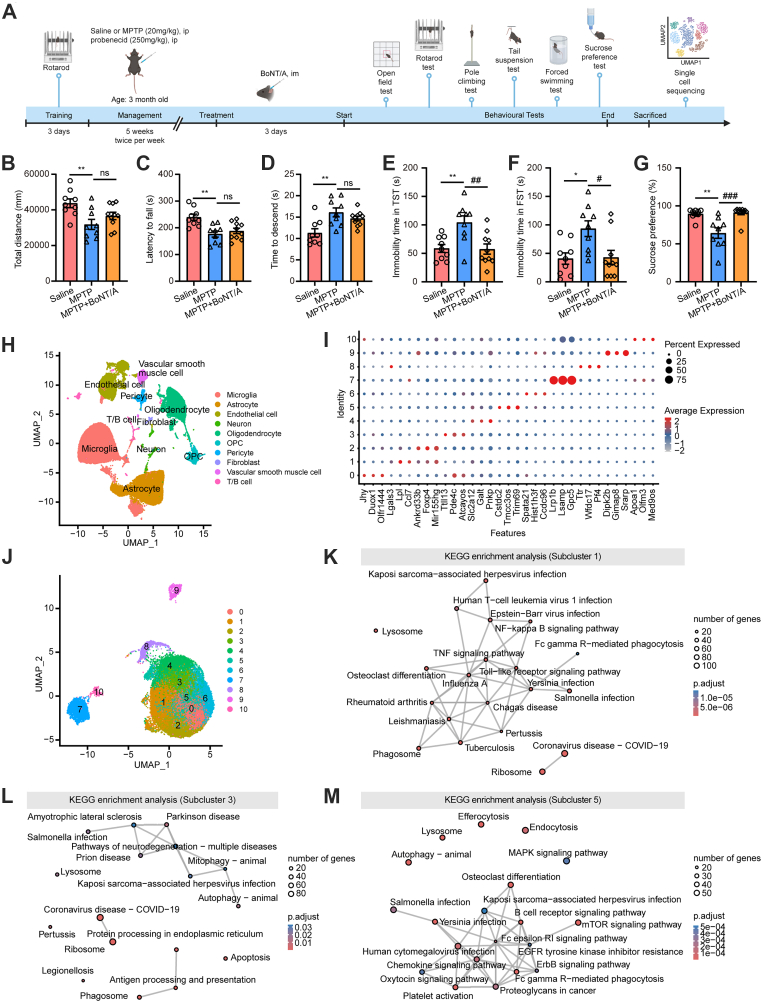
**Single-cell sequencing of the hippocampus in MPTP/probenecid-induced Parkinson's disease mice treated with Botulinum Neurotoxin A (BoNT/A). (A)** Timeline for the MPTP/probenecid-induced PD mice following BoNT/A treatment. C57BL/6J mice underwent MPTP/probenecid-induced PD treatment, receiving 20 mg/kg MPTP and probenecid (250 mg/kg, i.p.) via intraperitoneal injections twice weekly for five weeks. BoNT/A was then administered at 10 U/kg into the cheek for three consecutive days. Behavioural assessments, including open field, rotarod, pole climbing, TST, FST, and SPT, were conducted afterwards. Mice were euthanised at the end of the experiment for single-cell sequencing analysis. Created with Biorender.com. **(B)** The total distance travelled in the open field test. *n* = 9–10 mice/group. **(C)** The latency to fall in the rotarod test. *n* = 9–10 mice/group. **(D)** The time to descend in the pole climbing test. *n* = 9–10 mice/group. **(E)** Immobility time in the TST. *n* = 9–10 mice/group. **(F)** Immobility time in the FST. *n* = 9–10 mice/group. **(G)** Sucrose preference percentage in the SPT. *n* = 9–10 mice/group. **(H)** UMAP visualisation showed cell type annotation based on marker genes for ten major cell types: microglia, astrocytes, endothelial cells, neurons, oligodendrocytes, oligodendrocyte precursor cells (OPC), pericytes, fibroblasts, vascular smooth muscle cells, and T/B cells. **(I)** Dot plot showing gene marker expression in microglial sub-clusters. Dot size represents the percentage of cells expressing each marker, and colour intensity indicates average expression levels. **(J)** UMAP visualisation illustrating the distribution of cells within the microglia cluster, into 11 distinct microglial sub-clusters. **(K**–**M)** KEGG enrichment analysis identifies pathways enriched in the microglia subcluster 1 **(K)**, subcluster 3 **(L)**, and subcluster 5 **(M)**. Colours indicate the p-adjusted value, and dot sizes indicate the number of genes contributing to the enrichment of the term. All data are presented as the mean ± SEM. Significance was assessed by one-way ANOVA with Tukey's post hoc. versus Saline group: ∗*P* < 0.05, ∗∗*P* < 0.01. versus MPTP group: ^#^*P* < 0.05, ^##^*P* < 0.01, ^###^*P* < 0.001.

Recent clinical trials have shown that BoNT/A can be beneficial in treating patients with depression in PD.^60^, ^61^, ^62^ We further investigated whether this effect involves complement C3–C3aR-mediated microglial phagocytosis in an MPTP-induced mouse model (Fig. 7A). Interestingly, while the administration of BoNT/A after a 5-week chronic MPTP/probenecid injection did not produce significant improvements in motor dysfunction, as evidenced by no notable differences in total travelled distance in the open field test, latency to fall in the rotarod test, or descending time in the pole test (Fig. 7B–D). Specifically, we observed a marked reduction in immobility time during the FST and TST, along with restored sucrose preference following BoNT/A treatment in the MPTP-treated mice (Fig. 7E–G). This suggests that BoNT/A has a promising potential to alleviate depressive symptoms associated with PD.

**Supplementary Figure 5:**
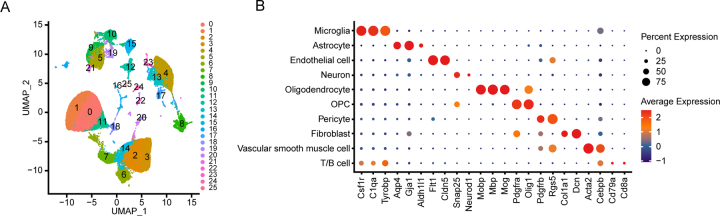
**Single-cell sequencing of the hippocampus in MPTP/probenecid-induced Parkinson's disease mice treated with Botulinum Neurotoxin A (BoNT/A). (A)** Uniform manifold approximation and projection (UMAP) visualisation is employed to analyse the clustering of single-cell transcriptomes from hippocampal cells derived from saline-treated, MPTP-lesioned, and MPTP-lesioned mice that received BoNT/A treatment. A total of 4 mice per group were included in the study. **(B)** Dot plot illustrates the relative expression levels of key canonical gene markers associated with prominent cell types, including microglia, astrocytes, endothelial cells, neurons, oligodendrocyte precursor cells (OPC), pericytes, fibroblasts, vascular smooth muscle cells, and T/B cells. The size of each dot reflects the proportion of cells within each cluster that express the respective marker genes, while the intensity of the dot's colour corresponds to the average expression level of these genes across the cell population.

To investigate the impact of BoNT/A on cellular interactions, we performed single-cell RNA sequencing (scRNA-seq) on hippocampal tissues obtained from Saline-, MPTP-, and MPTP+BoNT/A-treated mice. Following stringent quality control and data filtering, we identified 26 distinct cellular clusters through UMAP dimensionality reduction (Fig. S5A). To elucidate the alterations in major hippocampal cell types, we utilised established canonical gene markers for each cell type (Fig. S5B). With these markers, we successfully categorised 10 major cell types: microglia (clusters 0, 1, 6, 11, 18), astrocytes (clusters 2, 3, 7, 14), neurons (clusters 20 and 22), endothelial cells (clusters 5, 9, 10, 19, 21), vascular smooth muscle cells (cluster 15), pericytes (cluster 12), fibroblasts (cluster 24), oligodendrocytes (clusters 4, 13, 17, 23), oligodendrocyte precursor cells (OPCs) (cluster 8), and B/T cells (clusters 16 and 25) (Fig. 7H). To further elucidate the impact of BoNT/A on microglial functionality, we conducted a sub-clustering analysis aimed at pinpointing specific microglial subpopulations implicated in this process. Reclustering yielded 11 distinct microglial subclusters, each characterised by unique functional profiles based on the expression of subtype-specific genes (Fig. 7I and J). KEGG enrichment analysis identified three of these subclusters 1, 3, and 5 as phagocytosis-related microglia, characterised by enriched pathways including lysosome, phagosome, and FcγR-mediated phagocytosis pathways (Fig. 7K–M). Collectively, these findings imply a definitive role for BoNT/A in modulating microglial phagocytic activity.

**Fig. 8:**
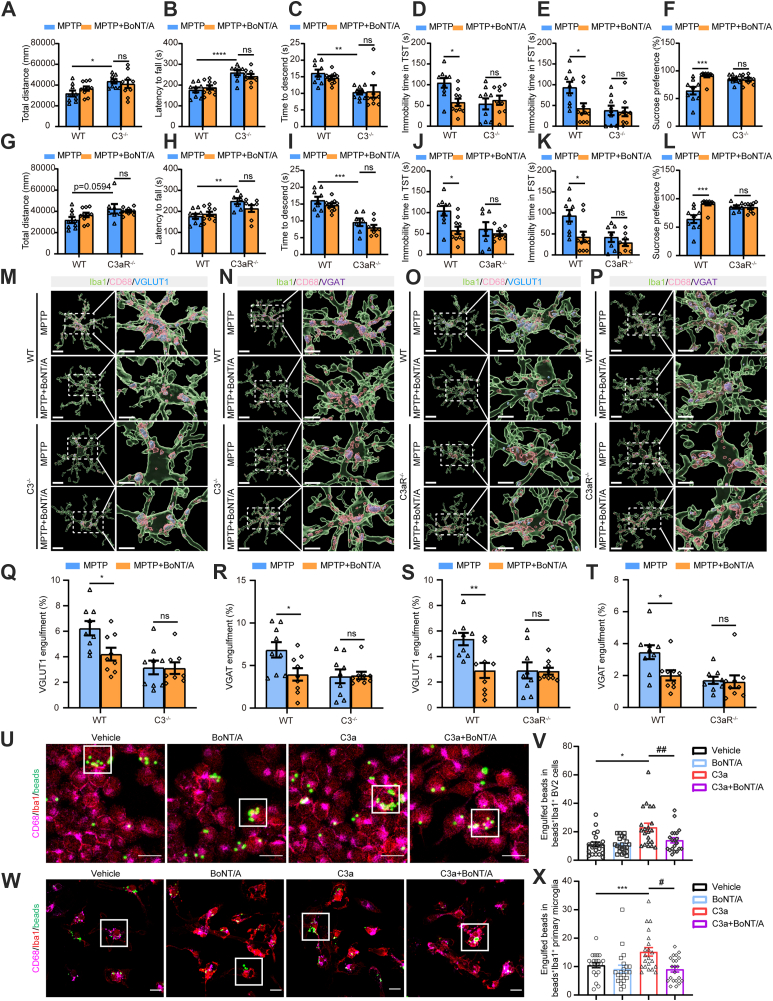
**Effect of BoNT/A on depressive-like phenotypes and microglial-mediated synaptic phagocytosis in MPTP/probenecid-treated mice lacking Complement *C3* and *C3aR*. (A**–**F)** The total distance travelled during the open field assessment **(A)**, the latency to fall in the rotarod test **(B)**, the time to descend in the pole climbing test **(C)**, immobility time in the TST **(D)**, immobility time in the FST **(E)**, the sucrose preference percentage in the SPT **(F)** were evaluated in *C3*-deficient mice with MPTP-induced PD post-BoNT/A administration (*n* = 8–10 mice/group). For *C3aR*-deficient mice **(G**–**L)**, similar assessments were conducted (*n* = 7–10 mice/group). **(M**–**N)** Representative Imaris images show microglial marker Iba1 (green), lysosomal marker CD68 (pink), and excitatory presynaptic marker VGLUT1 (blue) **(M)** or inhibitory presynaptic marker VGAT (purple) **(N)** in *C3*-deficient mice post-BoNT/A treatment. Scale bars: 10 μm (left), 5 μm (zoomed-in). Images for *C3aR*-deficient mice were similarly displayed **(O–P)**. Statistical analysis of phagocytosed synaptic VGLUT1 **(Q)** and VGAT volume **(R)** involved 9 images from 3 mice. Quantitative analysis for *C3aR*-deficient mice was also conducted **(S**–**T)**. **(U)** Representative images depicting the phagocytic uptake of latex beads by BV2 microglial cells following treatment with the C3aR agonist, C3a, in conjunction with BoNT/A. scale bar = 20 μm. **(V)** Quantification of the number of latex beads phagocytosed by BV2 cells (*n* = 21 images from 3 slides, with 7 images taken per slide). **(W)** Images illustrating the phagocytic activity of primary microglial cells towards latex beads after treatment with the C3aR agonist, C3a, in combination with BoNT/A. scale bar = 20 μm. **(X)** Assessment of the number of latex beads internalised by primary microglial cells (*n* = 21 images collected from 3 slides, with 7 images acquired per slide). All data are presented as the mean ± SEM. Significance was assessed by two-way ANOVA with Tukey's post hoc. ∗*P* < 0.05, ∗∗*P* < 0.01, ∗∗∗*P* < 0.001. versus C3a group: ^#^*P* < 0.05, ^##^*P* < 0.01.

To investigate the role of the complement C3–C3aR pathway in mediating the effects of BoNT/A in the MPTP-induced Parkinsonian mouse model, we evaluated the outcomes in both *C3* and *C3aR* knockout mice. Our results indicated that the absence of complement *C3* and *C3aR* led to an improvement in locomotion, as evidenced by the increased total distance travelled in the OFT, increased latency to fall in the rotarod test, and decreased descent time in the pole test. Notably, BoNT/A treatment provided no additional motor benefit (Fig. 8A–C, 8G-I). Conversely, treatment with BoNT/A significantly decreased immobility times in the TST and FST and improved sucrose preference in the SPT. However, these antidepressant effects of BoNT/A were abolished in the *C3* and *C3aR* knockout mice (Fig. 8D–F, J–L), suggesting that the antidepressant-like properties of BoNT/A depend on the complement C3–C3aR signalling pathway.

To evaluate whether the modulation of microglial phagocytic activity by BoNT/A is dependent on C3–C3aR signalling, we conducted 3D Imaris reconstructions of microglia that were co-immunostained for Iba1, CD68, and either VGLUT1 or VGAT. Our analysis demonstrated a significant decrease in the volume of VGLUT1^+^ or VGAT^+^ synaptic material being engulfed by CD68^+^ lysosomes in hippocampal microglia from BoNT/A-treated mice in comparison to the MPTP mouse model. Notably, these protective effects were completely abrogated in *C3* and *C3aR* knockout mice (Fig. 8M–T). To further explore the relationship between BoNT/A and the C3–C3aR complement pathway, we conducted an in vitro microglial phagocytosis assay. We treated BV2 and primary microglial cells with the C3aR agonist C3a, respectively, followed by BoNT/A treatment, and observed notable changes in the phagocytosis of latex beads by these cells. Our analysis involved quantifying the number of beads consumed by the most phagocytic BV2 and primary microglial cells (Fig. 8U–X). The results indicated that C3a significantly enhanced the phagocytic ability of microglial cells towards latex beads. However, the subsequent treatment with BoNT/A appeared to effectively counteract the stimulatory effects of C3a (Fig. 8U–X), suggesting that BoNT/A can inhibit the C3a-induced enhancement of phagocytosis in BV2 and primary microglial cells. This study enhances our comprehension of the interplay between BoNT/A and the complement pathway in modulating microglial activity. It demonstrates that the inhibitory effect of BoNT/A on microglial phagocytosis is contingent upon the functional integrity of the complement C3–C3aR signalling axis.

## Discussion

Depression is an early symptom or risk factor in PD, with prevalence estimates around 35%. A growing body of evidence highlights the significance of biological sex in the development and phenotypical expression of PD.^78^ Notably, men exhibit a twofold higher risk of developing PD compared to women.^79^ However, it is important to note that women experience a higher mortality rate and a faster disease progression.^80^ These differences suggest sex-specific pathogenic mechanisms, yet the underlying molecular basis remains poorly understood.

Although numerous proteomics studies on PD have been reported—involving clinical samples such as blood,^13^^,^^19^^,^^81^, ^82^, ^83^ urine,^84^ cerebrospinal fluid,^85^^,^^86^ and brain,^87^, ^88^, ^89^ as well as brain tissues from PD animal models.^52^^,^^90^ Among the blood-based candidate biomarkers identified through proteomics for PD across 12 studies,^12^^,^^91^^,^^92^ ApoA-I and haptoglobin were the most replicable, appearing in five cohorts. Other replicable biomarkers, including Clusterin, α2M, ApoM, ApoB, ApoA-IV, transthyretin, and ITIHC4, and several complement proteins such as complement C1Q, C1R, C3, C4, C5, and CFH were identified for diagnosis and prognosis of PD.^91^ However, research specifically targeting the proteomics of PD non-motor symptoms, particularly through a sex-specific lens, remains scarce.

In this study, we performed a comprehensive analysis of plasma samples from male and female patients with PD and those with depression (DPD), utilising data from the Parkinson's Progression Markers Initiative (PPMI) and an independent validation cohort. Based on the proteomic profiling of plasma from patients with PD comorbid with depressive symptoms in the PPMI dataset, we underscore the critical involvement of immune and inflammatory pathways in DPD and PD pathogenesis, with notable sex-specific regulation. Overall, differential protein expression between patients with DPD and PD highlights upregulated immune processes, including T cell, lymphocyte, and mononuclear cell proliferation, myeloid leukocyte activation, and glial cell activation. These findings collectively emphasise that immune dysregulation is a central feature of DPD. However, sex-stratified analysis revealed divergent immune modulation. In males, patients with DPD are associated with downregulation of immune activation, including negative regulation of lymphocyte/leukocyte activation and positive modulation of PI3K signalling. In contrast, females with DPD exhibit a pronounced up-regulation of immune activity, characterised by positive regulation of lymphocyte/leukocyte activation and small GTPase-mediated signalling pathways. These observations suggest that while both sexes share common alterations in immune cell proliferation and microglial activation, the direction of immune modulation—specifically regarding lymphocyte and leukocyte activation—diverges significantly between males and females.

Based on rigorous validation analysis in an independent clinical cohort, our present findings confirm that several critical DEPs in PD and DPD, including complement-related proteins CFH, C1R, A2M, and APOM, are highly consistent with previously reported biomarkers in relevant literature,^91^ further verifying that these proteins play fundamental and functionally conserved roles in the progression of PD and DPD. Subsequent comparative analysis of sex-stratified DEPs between male and female patients with PD, DPD, and healthy controls (HC) yielded a striking observation: while complement activation and the complement and coagulation cascades were consistently and significantly enriched in both male and female cohorts across PD versus HC and DPD versus HC comparisons, indicative of a core and trans-sexual pathological mechanism whereby complement pathway dysregulation deeply contributes to both PD pathogenesis and the development of depressive comorbidity (DPD), marked sexual dimorphism was evident in other dysregulated pathways and functional processes. Downregulated DEPs displayed minimal functional overlap between sexes, with female-specific enrichment linked to nucleoside triphosphate metabolism, central carbon metabolism, and multiple neurodegenerative pathways, whereas male downregulated DEPs were predominantly associated with lipoprotein and cholesterol remodelling. More notably, direct comparison of DEPs between DPD and PD revealed further pronounced sex disparities. Robust functional enrichment was exclusively detected in male DEPs, encompassing cellular homoeostasis, endoplasmic reticulum stress regulation, intestinal IgA-mediated immune networks, cardiac electrophysiological processes, and key signalling and cardiovascular pathways, highlighting gut immune dysfunction and systemic signalling disturbances as male-specific pathological features of DPD; by contrast, female DEPs showed no significant functional enrichment in this direct comparison, with only weak, isolated terms related to cell adhesion and tyrosine metabolism identified. Collectively, these data delineate both conserved complement-driven immune dysfunction and profound sex-divergent molecular perturbations underlying PD and DPD, emphasising marked sexual dimorphism in the pathophysiology of depressive comorbidity in PD that drives distinct disease heterogeneity between male and female patients.

In line with the critical involvement of complement pathway dysregulation in the progression of both PD and DPD across sexes, complement activation has been widely implicated in multiple PD models, including α-syn PFF–injected and A53T transgenic mice, which exhibit pronounced upregulation of complement and coagulation cascades.^52^ The C3/C3aR pathway governs astrocyte–neuron crosstalk and regulates α-synuclein pathology and neuronal survival through NF-κB and GSK3β signalling cascades.^93^^,^^94^ Furthermore, Complement C4 exacerbates α-synuclein aggregation, synaptic loss, and dopaminergic neurodegeneration in vivo.^95^ Additionally, microglial CR3 drives neuronal ferroptosis via NOX2-mediated iron accumulation in rotenone-induced PD models.^96^ PD is characterised by heightened activation of the complement pathway, which is intricately linked to microglial overactivation and disruptions in synaptic function.^97^ However, the role of complement-mediated microglial synaptic phagocytosis in the development of depression within PD remains unclear. Here, we utilised an MPTP/probenecid-induced mouse model that mimics PD alongside depressive-like behaviours and motor deficits. Mass spectrometry analysis of brain regions revealed that complement component C3 was notably upregulated in the hippocampus, which is consistent with previous α-Synuclein-Based Mouse Models of PD.^52^ MPTP-treated male and female mice exhibited increased levels of hippocampus and mPFC complement components (C1Q, C3, C3aR) and related downstream signalling (p-STAT3, p-P65), accompanied by microglial activation, synaptic loss, and elevated microglial engulfment of synaptic material. Importantly, the genetic deletion of *C3* in MPTP mice led to significant improvements, rescuing motor deficits and depressive-like behaviours, while also preventing synaptic loss and microglial engulfment. These findings underscore the potential for complement C3 as a therapeutic target in addressing both motor and mood-related aspects of PD.

There are few controlled trials assessing antidepressants within the PD population, despite the frequent occurrence of depression among these patients and their use of antidepressants. In general, all traditional antidepressants and some dopamine agonists have proven to be both safe and well-tolerated for alleviating depressive symptoms in PD,^98^ even in light of earlier concerns regarding potential worsening of parkinsonism.^99^ Current pharmacological treatments for depression in PD primarily include selective serotonin reuptake inhibitors (SSRIs), serotonin and norepinephrine reuptake inhibitors (SNRIs), and tricyclic antidepressants. While the efficacy of these antidepressants in PD shows some variability based on the time course of response, pooled analyses indicate a moderate benefit.^22^^,^^99^^,^^100^ Botulinum Neurotoxin A (BoNT/A) is a highly effective neurotoxin that has found diverse applications beyond movement disorders,^101^^,^^102^ extending into areas such as limb spasticity,^103^ pain management,^104^ and autonomic dysfunction.^105^^,^^106^ There are ongoing explorations into its potential use for conditions like depression.^33^^,^^107^, ^108^, ^109^ Our preliminary research indicates that periocular injections of Botulinum Neurotoxin A into the facial expression muscles can significantly improve depressive symptoms in patients with PD, showing comparable results to sertraline.^62^ Notably, this approach demonstrated longer-lasting effects, a lower incidence of adverse reactions, and a high safety profile. Therefore, it presents a potential therapeutic option for individuals facing the dual challenges of PD and depression. Recently, accumulating evidence from our group and others has demonstrated that BoNT/A exerts antidepressant effects not only in PD-related depression but also in general depression models, indicating its broad antidepressant properties rather than disease-specific action. Our findings suggest that BoNT/A can be beneficial in improving depressive-like behaviours and addressing synaptic loss associated with microglial engulfment triggered by the classical complement pathway.^68^ Furthermore, BoNT/A shows potential to reduce proinflammatory responses mediated by microglia in a reserpine-induced mouse model of PD. However, whether and how BoNT/A regulates complement-mediated microglial synaptic phagocytosis to exert its antidepressant efficacy in PD-associated depression remains poorly defined. In this study, we further highlighted the therapeutic potential of BoNT/A in alleviating depressive-like behaviours in MPTP mouse models using single-cell RNA sequencing. Our findings demonstrate that BoNT/A has a positive effect on specific microglial subclusters associated with phagocytosis. Notably, the antidepressant effects of BoNT/A, along with its capacity to reduce microglial synaptic engulfment, were shown to depend on a functional C3–C3aR signalling pathway. This was substantiated by the absence of these effects in mice lacking either *C3* or *C3aR*, revealing that the conserved complement-dependent neuroimmune mechanism underlies the antidepressant action of BoNT/A in PD-associated depression, underscoring the essential role of this signalling pathway in mediating the beneficial outcomes of BoNT/A. An important mechanistic question concerns how peripherally injected BoNT/A can modulate central hippocampal microglia. Although administered into facial muscles, BoNT/A may exert central effects through retrograde axonal transport and interneuronal transcytosis, as supported by recent studies. Ewa Bomba-Warczak et al. showed that BoNT/A undergoes retrograde transport in non-acidified organelles and interneuronal transcytosis, propagating its active form across neural circuits to mediate long-distance effects.^110^ Consistently, Ni et al. confirmed that peripherally injected BoNT/A is retrogradely transported to facial motoneuron somata and further transcytosed to second-order neurons, thereby modulating mood-related central circuits.^111^ While the precise route remains to be fully defined, our data demonstrate that BoNT/A ultimately inhibits C3–C3aR-dependent microglial synaptic pruning in the hippocampus, independent of its peripheral motor effects.

While this study provides compelling evidence for sex-specific proteomic mechanisms and the critical role of complement-mediated synaptic pruning in patients with Parkinson's disease and comorbid depression (DPD), several important limitations warrant explicit acknowledgement.

First, our clinical proteomic analyses in both the PPMI discovery cohort and independent validation cohort were constrained by relatively small sample sizes within the DPD subgroup, a challenge directly driven by well-documented sex disparities in disease epidemiology during participant recruitment: Parkinson's disease is more prevalent in males, while depressive symptoms are more common in females, making it difficult to enrol balanced numbers of male and female patients with PD and comorbid depression. In addition, all participants in the local validation cohort were East Asian individuals from the Yangtze River Delta region of China, which provides geographic and ethnic homogeneity but may limit broader applicability. This inherent recruitment constraint inevitably reduces the generalisability of our sex-stratified proteomic findings. Future studies with expanded sample sizes, balanced sex distribution, and multi-centre and multi-ethnic enrolment are therefore warranted to further validate and extend these preliminary observations and to evaluate the generalisability of the present findings across diverse racial, ethnic, and geographic populations. Second, in the MPTP/probenecid mouse model, potential confounding effects of locomotor impairment on the interpretation of depressive-like behaviours in the forced swimming test and tail suspension test cannot be fully excluded. Emerging circuit-based evidence also supports overlapping neural substrates for motor and non-motor symptoms in PD,^29^ suggesting intrinsic interconnections between these phenotypes. Although we included the sucrose preference test, a non-locomotor measure of anhedonia, to reduce motor confounds, overlapping neural substrates for motor and non-motor symptoms in PD may still affect behavioural interpretation. Third, all in vivo mechanistic experiments were exclusively performed using male *C3* and *C3aR* knockout mouse models, which creates a notable translational limitation given the prominent sexual dimorphism observed clinically in DPD pathophysiology. Although male-only modelling was adopted to exclude confounding hormonal effects of oestrogen, this design inherently restricts the generalisability of our mechanistic findings to female populations. Finally, although BoNT/A significantly alleviated depressive-like behaviours in our preclinical models, the precise molecular cascade underlying its modulation of the C3–C3aR signalling axis in microglia, including direct versus indirect regulatory effects and downstream effector pathways, remains incompletely defined. Future studies using microglia-specific conditional knockout models will be critical for further dissecting the cell-type-specific roles of the C3/C3aR axis and fully delineating the therapeutic mechanism of BoNT/A. Addressing these limitations will be critical for validating our findings in larger, multi-centre clinical cohorts with balanced sex distribution and validating mechanistic conclusions in female animal models to advance the translational potential of complement-targeted therapies for DPD.

In conclusion, this study demonstrates that depression in Parkinson's disease (DPD) is underpinned by significant sex-specific immune dysregulation and conserved complement activation. A convergent finding across sexes is the critical role of the complement system, specifically the C3–C3aR axis. Notably, all key mechanistic experiments, including genetic knockout of C3/C3aR and BoNT/A intervention, were performed exclusively in male mice; whether the same mechanism operates in female mice remains to be investigated. In a MPTP/probenecid-induced mouse model, this pathway drives microglial phagocytosis of synapses in the hippocampus, leading to depressive-like behaviours. The therapeutic effect of BoNT/A in alleviating these depressive symptoms is mechanistically dependent on its ability to suppress this complement-mediated microglial synaptic pruning in male mice. These findings advocate for sex-stratified diagnostic approaches and identify the C3–C3aR pathway as a promising target for personalised therapy in Parkinson's disease with depression.

## Contributors

Qiao Yin: Data Curation; Formal Analysis; Investigation; Methodology; Visualisation; Writing–Original Draft Preparation. Mengyang Ding: Data Curation; Formal Analysis; Investigation; Methodology; Visualisation; Writing–Original Draft Preparation. Yurui Tang: Data Curation; Formal Analysis; Investigation; Methodology; Visualisation; Writing–Original Draft Preparation.Yuwan Qi: Data Curation; Formal Analysis; Investigation. Yuan Qin: Data Curation; Formal Analysis; Investigation; Methodology; Software; Writing–Original Draft Preparation. Hong Jin: Formal Analysis; Funding Acquisition; Investigation; Resources. Yang Li: Formal Analysis; Funding Acquisition; Investigation; Resources. Jili Bao: Investigation. Shuyang Ma: Investigation. Ying Li: Investigation. Haozhe Ding: Investigation. Xinyu An: Investigation. Enyou Qiao: Investigation. Yan Tang: Investigation. Qilin Zhang: Investigation. Linna Wang: Investigation. Jianfeng Shao: Investigation. Jianfeng Feng: Software; Resources. Li-Fang Hu: Conceptualisation; Resources; Writing–Review & Editing. Jing Wang: Conceptualisation; Funding Acquisition; Project Administration; Supervision; Writing–Review & Editing. Pan Fang: Conceptualisation; Funding Acquisition; Project Administration; Supervision; Writing–Review & Editing. Weifeng Luo: Conceptualisation; Funding Acquisition; Resources; Supervision. Qifei Cong: Conceptualisation; Funding Acquisition; Project Administration; Supervision; Writing–Original Draft Preparation; Writing–Review & Editing. Qiao Yin, Mengyang Ding, Yurui Tang, Yuwan Qi, and Yuan Qin have accessed and verified the underlying data. All authors have read and approved the final version of the manuscript prior to submission.

## Data sharing statement

Single-cell RNA sequencing data have been archived in the Gene Expression Omnibus (GEO) under accession number GEO: GSE308594. All raw mass spectrometry data have been submitted to the iProX partner repository with the dataset identifier IPX0016379000. The data will be available for open access upon publication. All the data used in the first-step study are available in the PPMI database (http://www.ppmi-info.org/data). Any information required for analysing the reported data is available upon request.

## Declaration of interests

Linna Wang is employed by Lanzhou Biotechnique Development Co., Ltd. The authors declare no other competing interests.

## Appendix A Supplementary data

Supplementary Table 1

Western Blots

STR profiling of cell lines

Mycoplasma testing
