# Supplementary figures and images for: The complement C3-microglial axis in depression of Parkinson's disease: from mechanism to therapeutic intervention
Source: eBioMedicine. 2026 Jun 9;129:106325. doi: 10.1016/j.ebiom.2026.106325 (PMC13273220; doi:10.1016/j.ebiom.2026.106325)

Figure 4I:


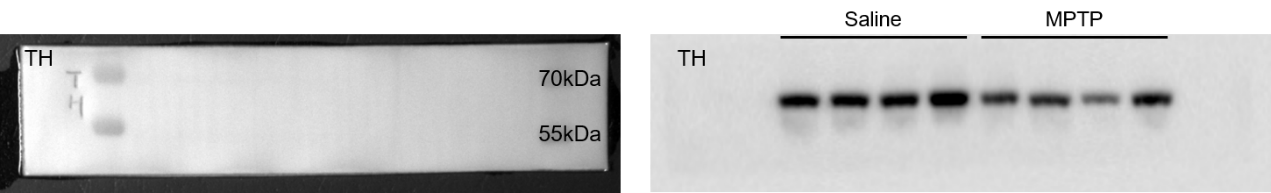

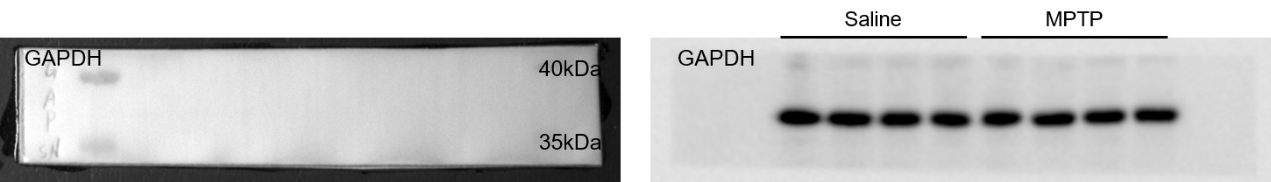


Figure 5A:


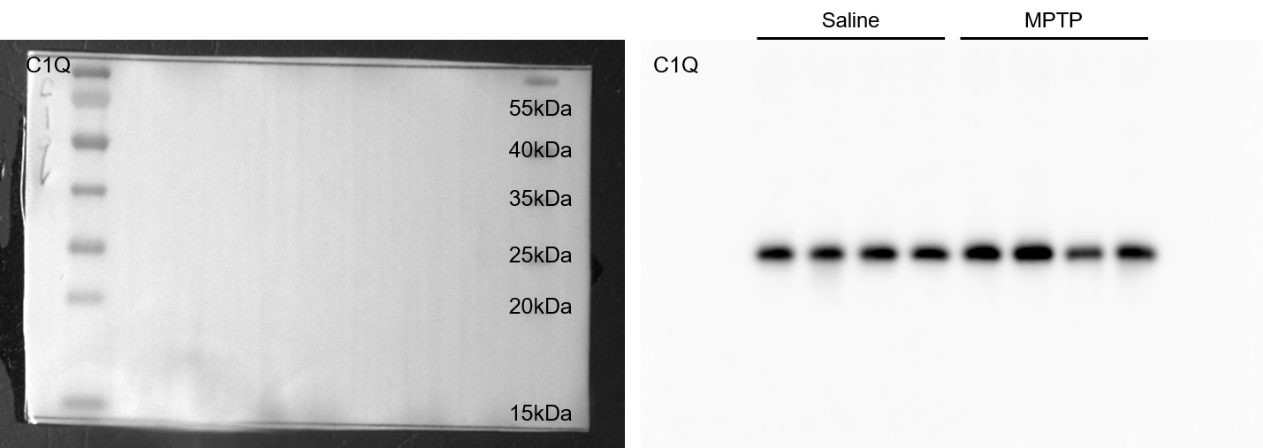

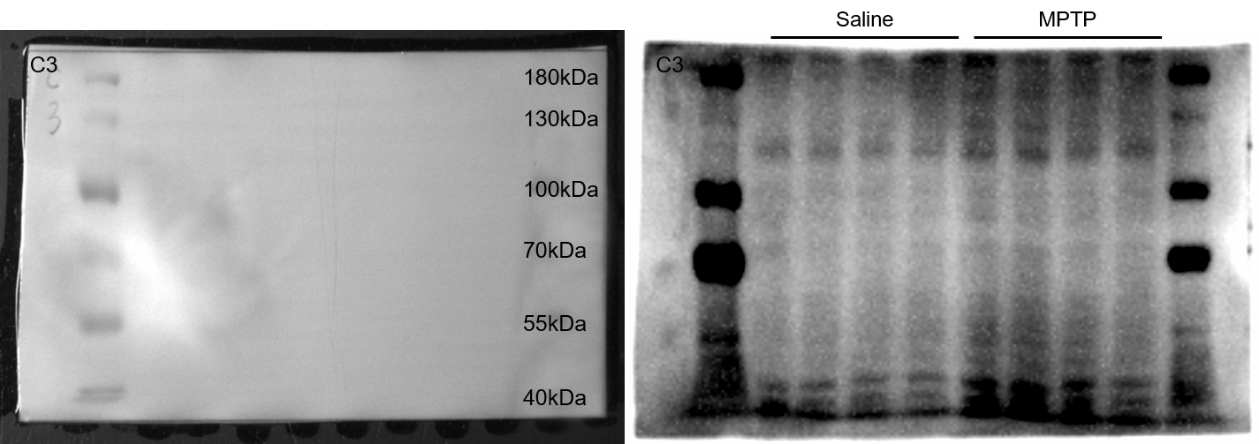

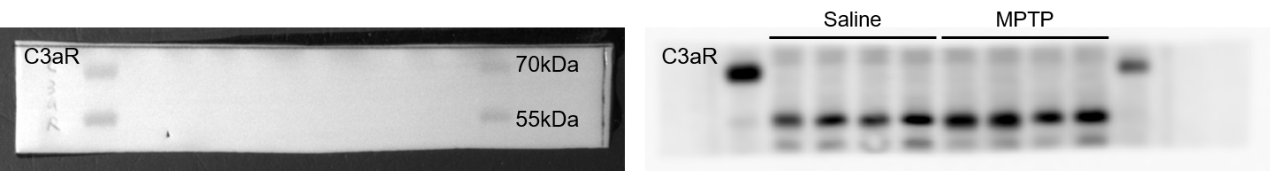


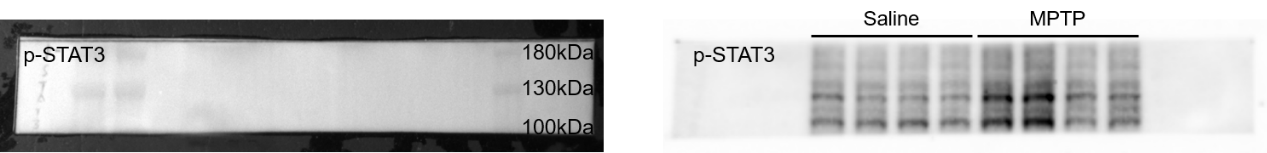

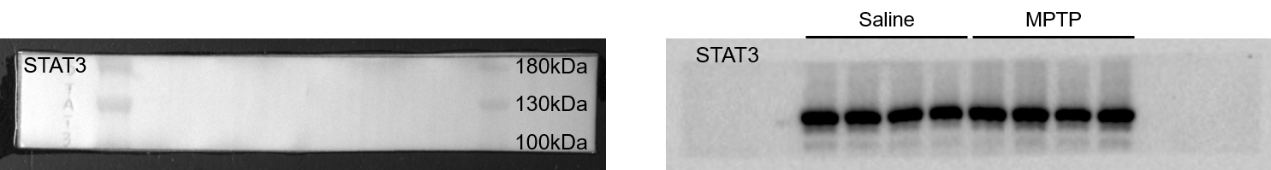


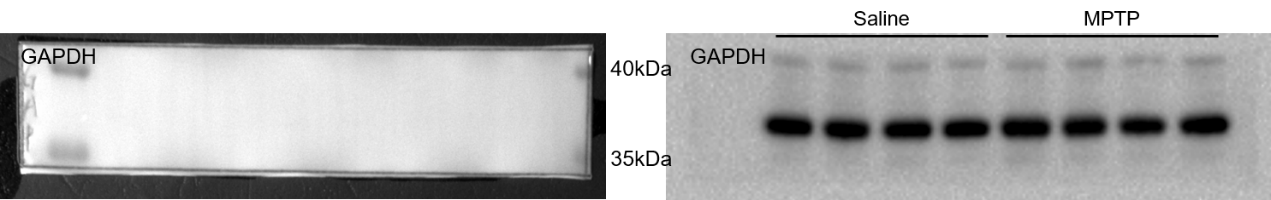


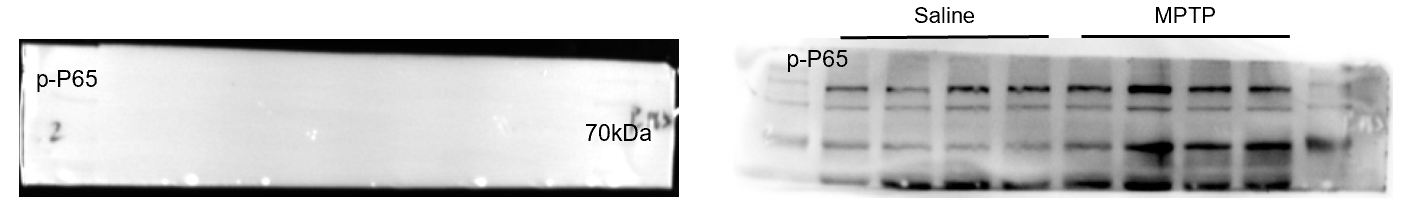


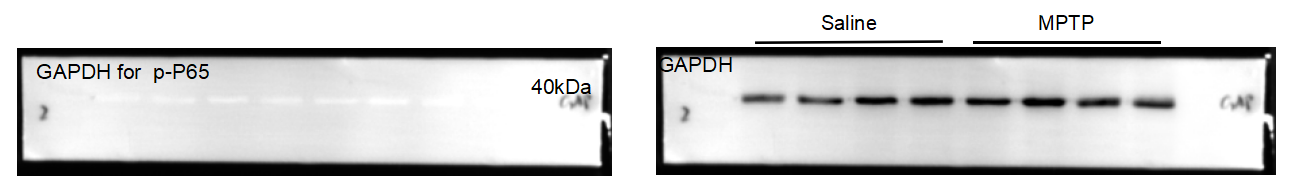


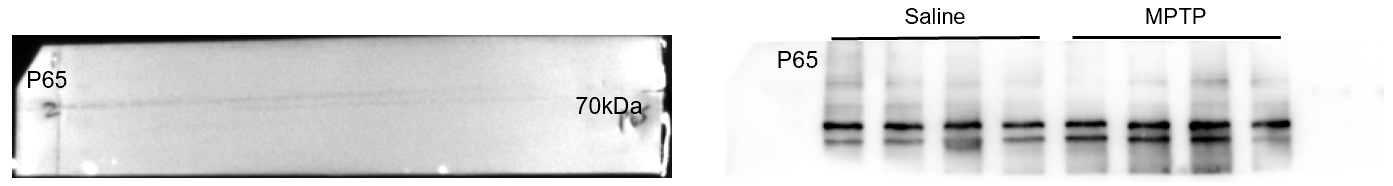


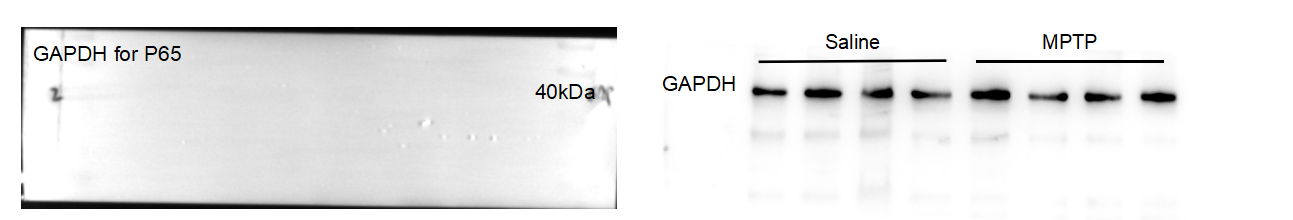


Supplementary Figure 4A:


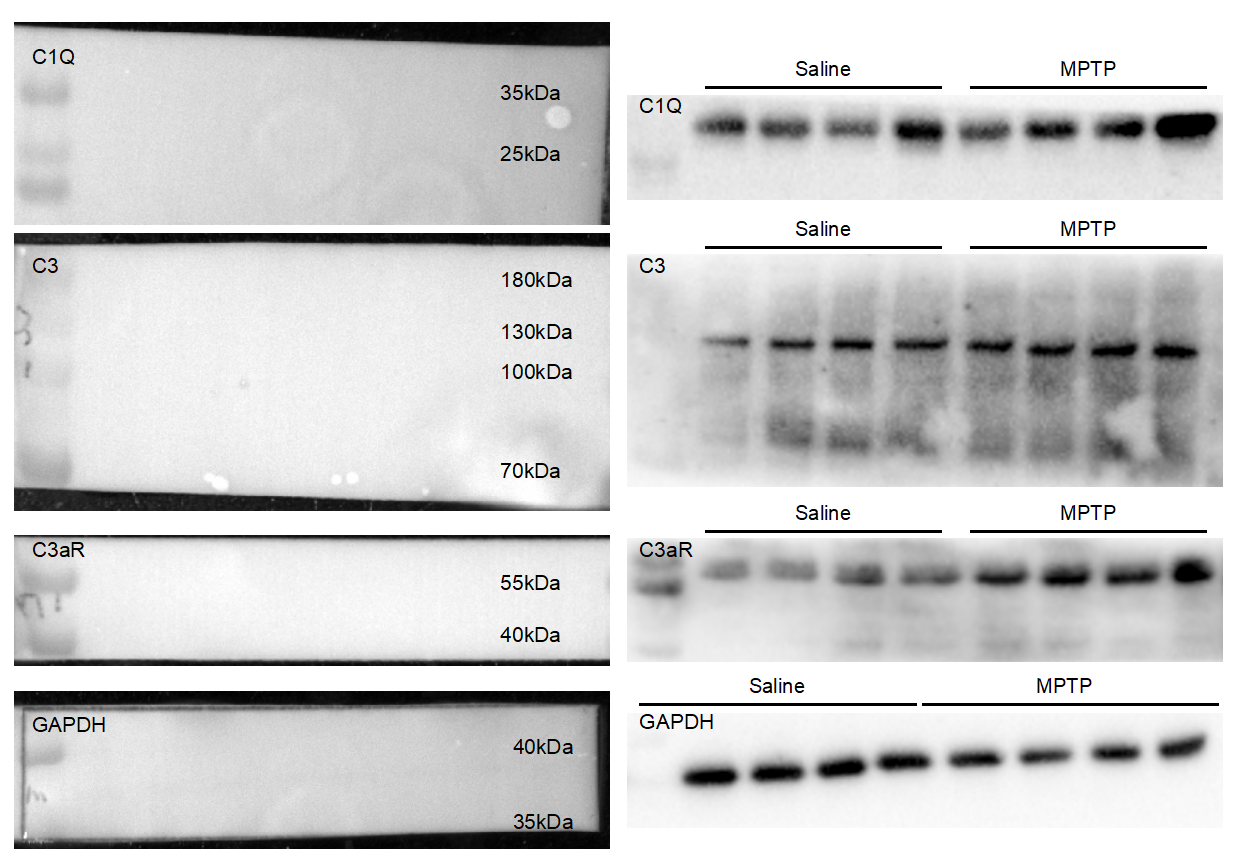
 Supplementary Figure 4E:


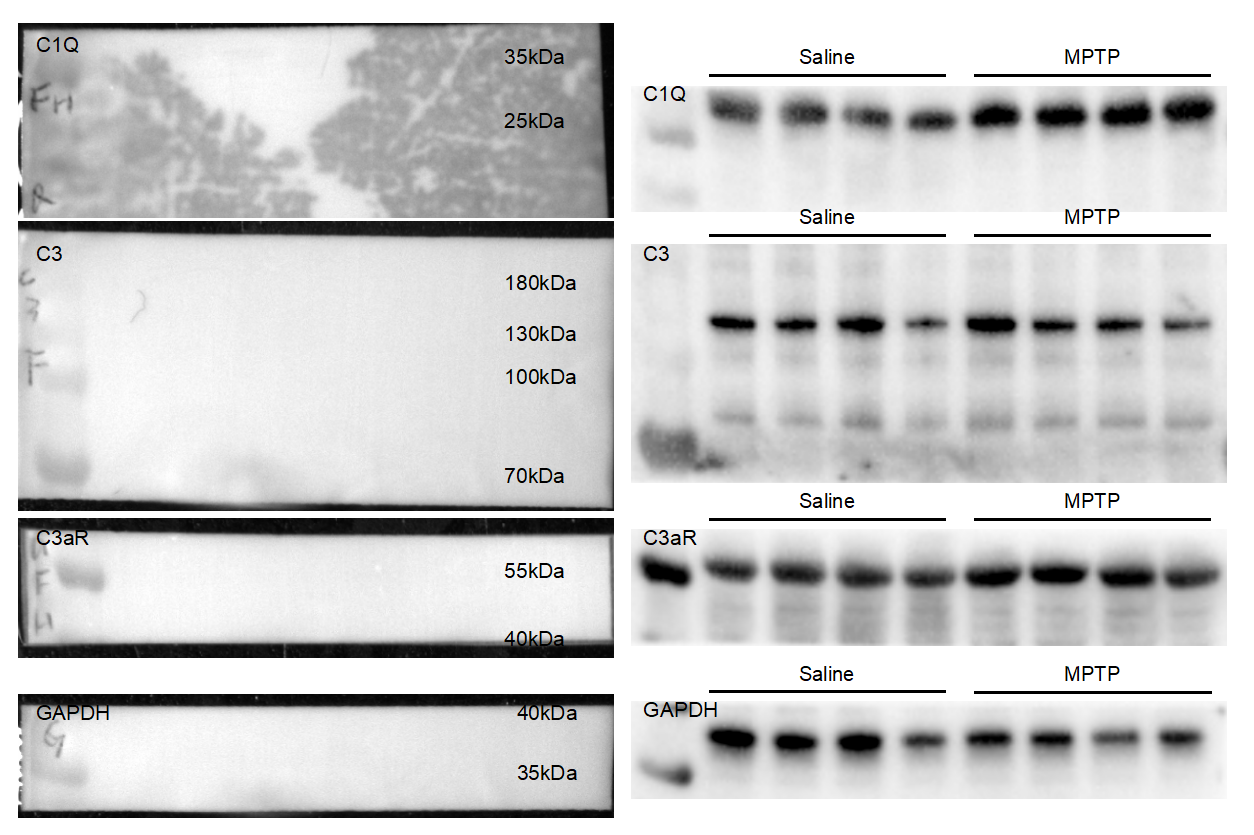


Supplementary Figure 4I:


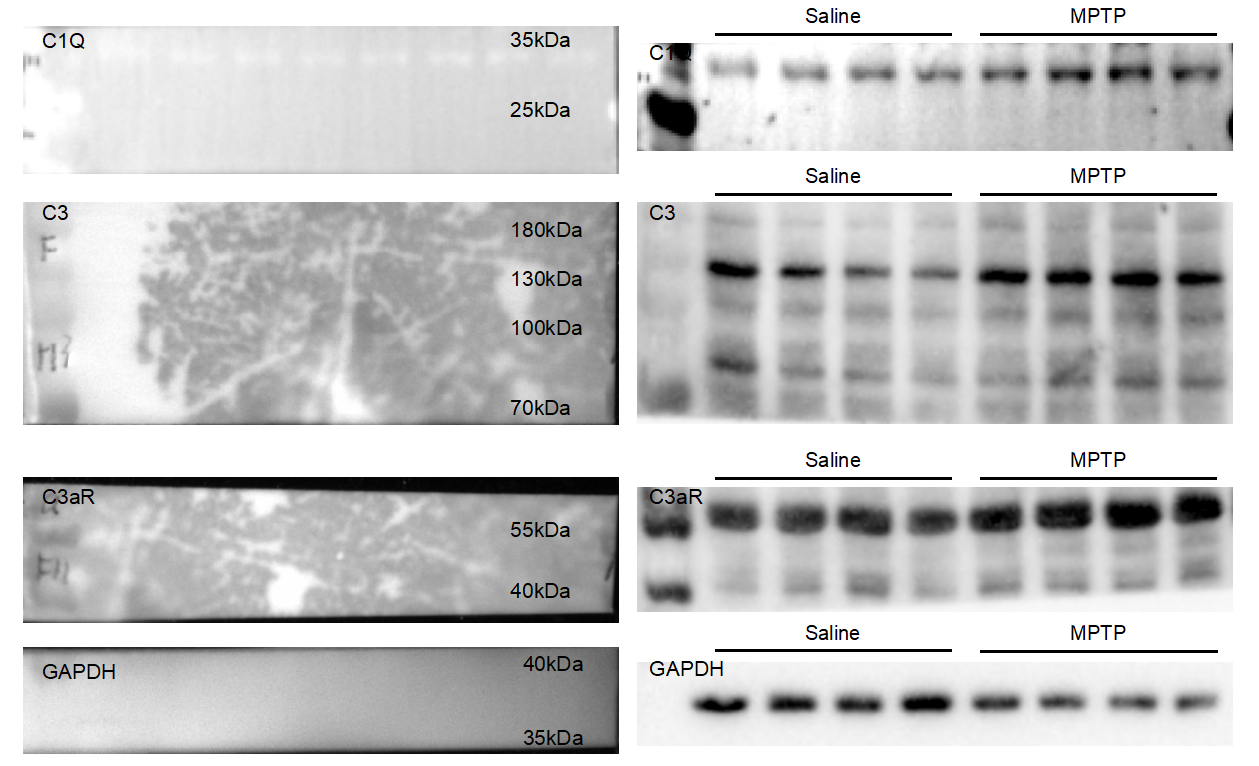

Supplement: Western Blots [file mmc2.docx]
