# Supplementary material for: The complement C3-microglial axis in depression of Parkinson's disease: from mechanism to therapeutic intervention
Source: eBioMedicine. 2026 Jun 9;129:106325. doi: 10.1016/j.ebiom.2026.106325 (PMC13273220; doi:10.1016/j.ebiom.2026.106325)

细胞遗传质量鉴定检测

Cell Line Authentication Service

STR 基因型检测报告

检测单位：上海葵赛生物科技有限公司

检品名称： 细胞系

报告日期： 2025/10/21

报 告 说 明

1. 本报告只对送检的来样负责。
2. 检验报告上的检验结果和检验单位名称，未经同意不得用于广告、评优及商业宣传。
3. 对本报告有异议，请于收到报告之日起十五日内以书面方式提出，逾期不予受理。
4. 对纸质检验报告涂改、增删，或未加盖检验单位印章的复印件均无效。

上海葵赛生物科技有限公司

样 品 信 息

样品编号：

客户样本编号	公司编号
BV2	2025-10-20-yw

样品数量： 1

样品性状： 细胞系

检测项目： STR

检测方法： 用 CWBIO Magbead Micro Sample DNA Kit 试剂盒(货号：CW3064S)提取 DNA，采用 20- STR 扩增方案扩增，使用小鼠 STR 分型试剂盒-18 位点(荧光 PCR-毛细管电泳法)在 ABI 3130XL 型遗传分析仪上对 STR 位点进行检测。

上海[illegible]赛生物科技有限公司

检 测 结 果

(一) 检验基本情况

编号	多等位基因	匹配细胞系	人源污染	与对比细胞匹配度 EV 值	匹配说明
2025-10-20-yw	否	BV2	无	0.978	基本匹配

样本基因型检验结果

- 多等位基因指三等位及以上基因现象。
- 本次检测各细胞分型结果良好。

(二) 各样本描述

- 2025-10-20-yw：该株细胞鉴定结果为**小鼠细胞系**，细胞 STR 分型结果与 CCRID 数据库细胞 **BV2** 细胞系基因型一致。STR 分型结果**基本匹配。** 本次检测在该细胞系中**未发现多等位基因，无交叉污染，无人源污染。**
- 备注：下列位点中 TH01 和 D5S818 为人源位点，用于检测该细胞是否有人源污染。

上海莱赛生物科技有限公司

（三）样本分型结果

细胞 2025-10-20-yw 的 STR 位点和 Amelogenin 位点的基因分型结果

Loci	送检细胞 STR 信息				细胞库细胞 STR 信息		
	送检细胞名：BV2				细胞库细胞名：BV2		
	Allele1	Allele2	Allele3	Allele4	Allele1	Allele2	Allele3
18-3	16	17			16	17	
4-2	20.3				20.3		
6-7	15				15		
19-2	13				13		
1-2	16.3	19			19		
7-1	26.2				26.2		
8-1	16				16		
1-1	16	17			16	17	
3-2	14				14		
2-1	16				16		
15-3	22.3	23.3	24.3		22.3	23.3	24.3
6-4	18				18		
13-1	17.1				17.1		
11-2	16				16		
TH01							
D5S818							
17-2	15				15		
12-1	17				17		
5-5	17				17		
X-1	27				27		

其 他 说 明

（一） 分型方案及位点分布

	方案 1	方案 2	方案 3	方案 4
1	18-3	1-2	2-1	TH01
2	4-2	7-1	15-3	D5S818
3	6-7	8-1	6-4	17-2
4	19-2	1-1	13-1	12-1
5		3-2	11-2	5-5
6				X-1

实验方案及位点

上海莱赛生物科技有限公司

签发日期：2025 年 10 月 21 日

Sample Name	SQ
Quicell-BV2	

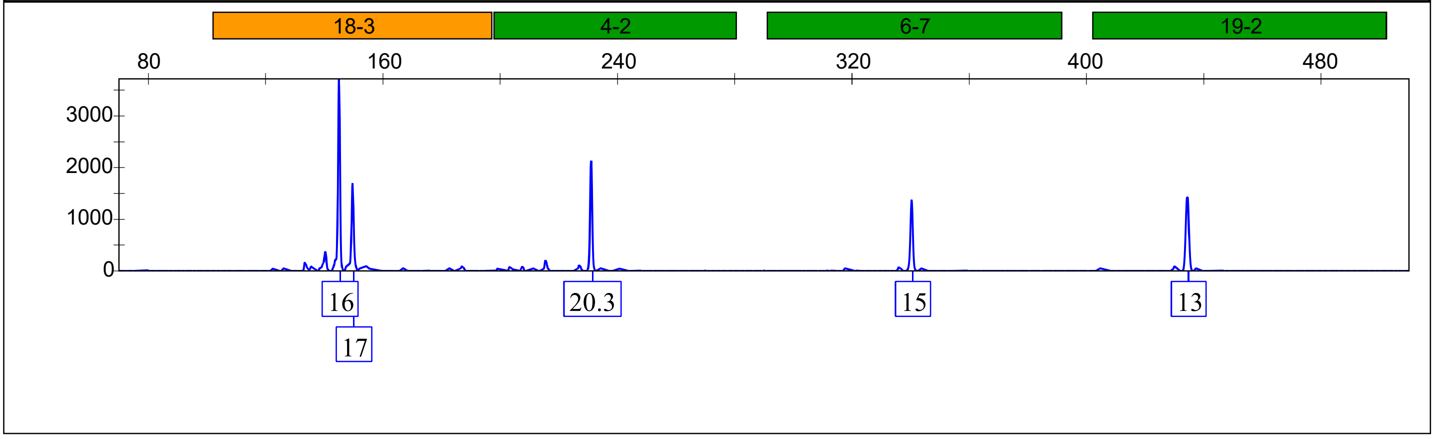

Quicell-BV2

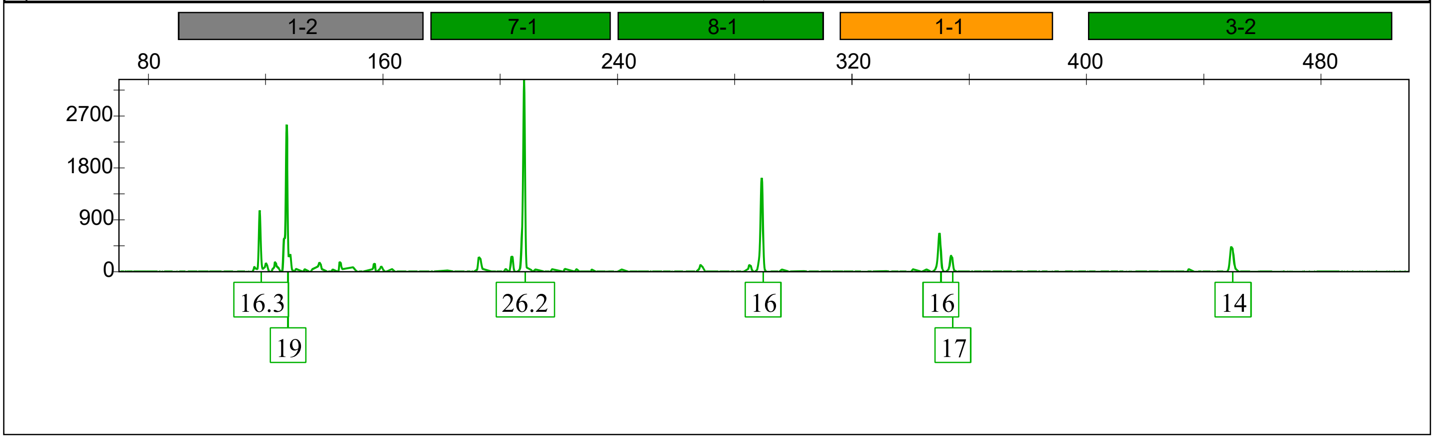

Quicell-BV2

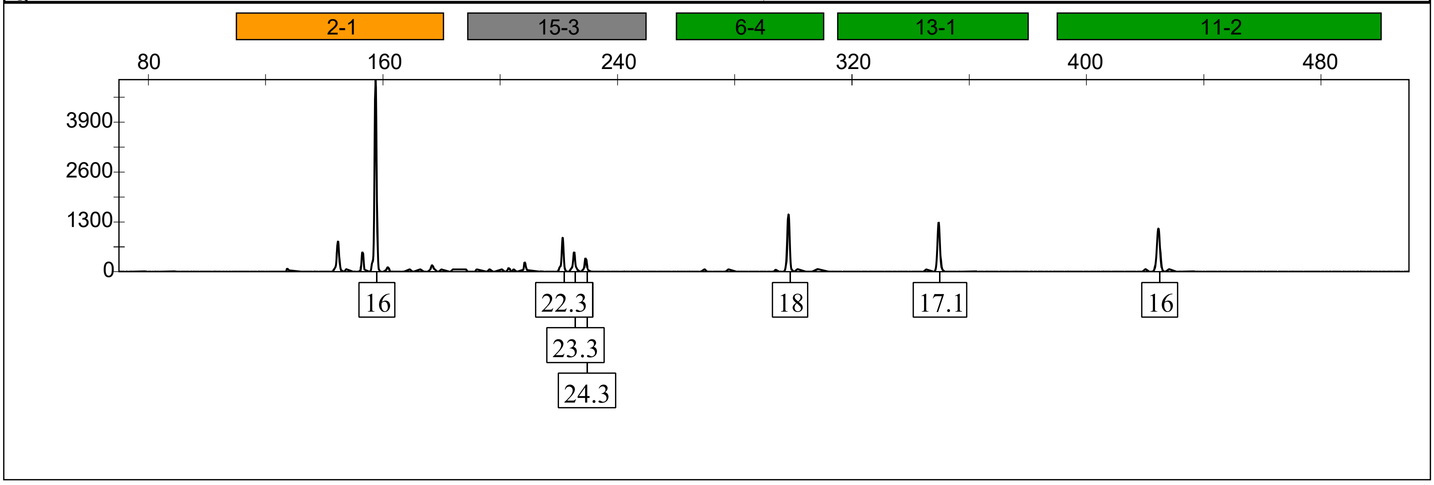

Quicell-BV2

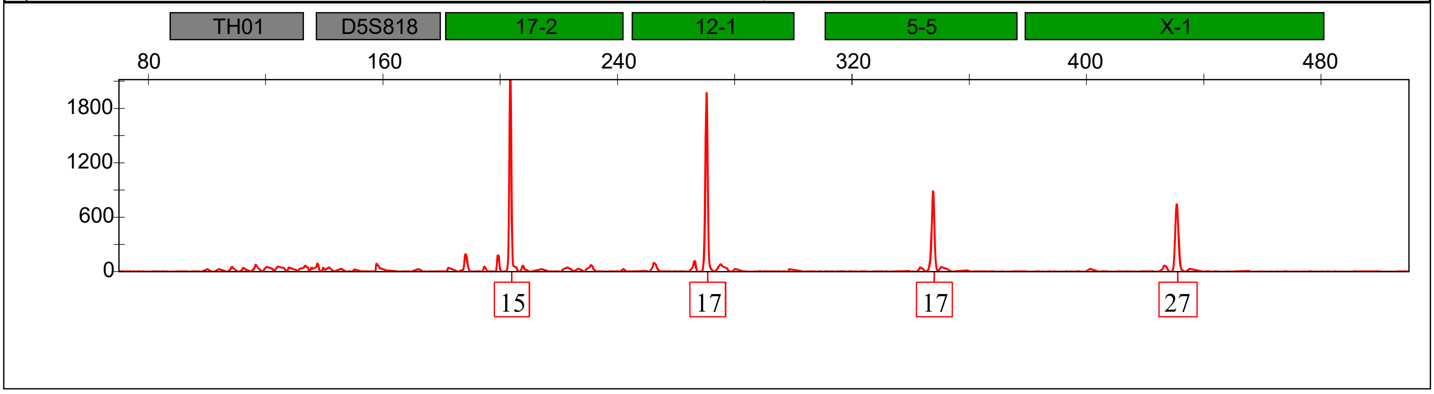

Supplement: STR profiling of cell lines [file mmc3.pdf]
