# Supplementary material for: The complement C3-microglial axis in depression of Parkinson's disease: from mechanism to therapeutic intervention
Source: eBioMedicine. 2026 Jun 9;129:106325. doi: 10.1016/j.ebiom.2026.106325 (PMC13273220; doi:10.1016/j.ebiom.2026.106325)
Supplement: Mycoplasma testing [file mmc4.pdf]

BV2 支原体检测报告

1．试验所需仪器设备及试剂

（1）仪器

仪器名称	规格型号	厂家
生物安全柜	BSC-1500IIA2-X	济南鑫贝西生物技术有限公司
CO_2 细胞培养箱	BC-J160S	上海博迅实业有限公司
凝胶成像系统	4600SF	Tanon
高速冷冻离心机	Multifuge X1R	Thermo Fisher
PCR 仪	FQD-96A	杭州博日科技股份有限公司
电泳仪电源	EPS300	Tanon
小型水平电泳槽	L-HET-7MN	LABGIC

（2）试剂耗材

试剂名称	规格 / 货号	厂家
支原体 PCR 检测试剂盒	BL1469A	Biosharp

2．支原体检测反应体系

试剂名称	用量
待检测细胞上清液	1ul
Mycoplasma PCR Mix（2×）	10ul
Mycoplasma Primer Mix	2ul
Mycoplasma Free Water	7ul

按照以上反应比例，在 PCR 管中将待检测样品上清液 1ul 加入到 19ul Mycoplasma PCR Mix 中，每次检测使用 Positive control 模版作为阳性对照，用 Negative control 模版作为阴性对照。

3．PCR 程序设置

步骤	温度	持续时间	循环数
预变性	98 ℃	2 min	1
变性	98 ℃	20 s	30
退火	56 ℃	25 s	
延伸	72 ℃	10 s	
	72 ℃	5 min	1

待检测样品中如果出现与阳性对照大小一致（约 500bp）的条带，说明样品细胞被支原体感染，若无对应条带则说明无支原体污染。

4．凝胶电泳结果

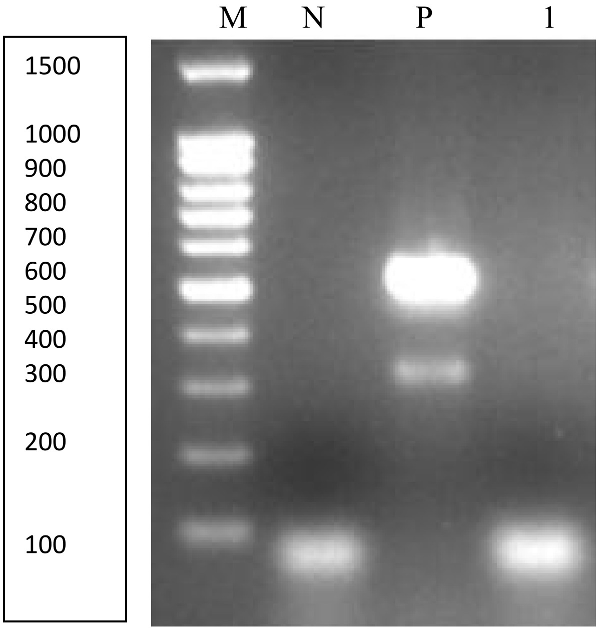

M 孔道为 DNA Marker
N 孔道为 Negative control
P 孔道为 Positive control
1 号孔道为待测 BV2 细胞样品

细胞结论：由电泳图可知检测的 BV2 细胞样品无支原体污染。

（注：图谱中样品点样孔最下方的条带是引物二聚体，是 PCR 检测时的常规现象，其亮暗程度通常是与阳性条带的亮度成反比的。PCR 的检测方法非常灵敏，一般极弱的阳性也可以看到有区别于二聚体的条带产生，结果可靠。）

检测人签名：王嘉禾
核验人签名：何恒
日期：25.10.21
